# Getting the biggest birch for the bang: restoring and expanding upland birchwoods in the Scottish Highlands by managing red deer

**DOI:** 10.1002/ece3.548

**Published:** 2013-05-22

**Authors:** Andrew J Tanentzap, James Zou, David A Coomes

**Affiliations:** 1 Forest Ecology and Conservation Group, Department of Plant Sciences, Downing Street, University of CambridgeCambridge, CB2 3EA, UK; 2 School of Engineering and Applied Sciences, Harvard UniversityCambridge, Massachusetts, 02138

**Keywords:** Afforestation, disturbance, evidence-based conservation, herbivory, modeling, restoration, wildlife management

## Abstract

High deer populations threaten the conservation value of woodlands and grasslands, but predicting the success of deer culling, in terms of allowing vegetation to recover, is difficult. Numerical simulation modeling is one approach to gain insight into the outcomes of management scenarios. We develop a spatially explicit model to predict the responses of *Betula* spp. to red deer (*Cervus elaphus*) and land management in the Scottish Highlands. Our model integrates a Bayesian stochastic stage-based matrix model within the framework of a widely used individual-based forest simulation model, using data collected along spatial and temporal gradients in deer browsing. By initializing our model with the historical spatial locations of trees, we find that densities of juvenile trees (<3 m tall) predicted after 9–13 years closely match counts observed in the field. This is among the first tests of the accuracy of a dynamical simulation model for predicting the responses of tree regeneration to herbivores. We then test the relative importance of deer browsing, ground cover vegetation, and seed availability in facilitating landscape-level birch regeneration using simulations in which we varied these three variables. We find that deer primarily control transitions of birch to taller (>3 m) height tiers over 30 years, but regeneration also requires suitable ground cover for seedling establishment. Densities of adult seed sources did not influence regeneration, nor did an active management scenario where we altered the spatial configuration of adults by creating “woodland islets”. Our results show that managers interested in maximizing tree regeneration cannot simply reduce deer densities but must also improve ground cover for seedling establishment, and the model we develop now enables managers to quantify explicitly how much both these factors need to be altered. More broadly, our findings emphasize the need for land managers to consider the impacts of large herbivores rather than their densities.

## Introduction

Increasing deer populations threaten the conservation of many different habitats, but predicting their long-term impacts and the outcomes of management actions to control their populations is difficult. High levels of deer browsing alter the structure and composition of plant communities and can prevent the regeneration of browse-intolerant plants in woodlands and grasslands (Fuller and Gill 2001; Côté et al. 2004; Takatsuki 2009). Changes to plant communities can have cascading effects on native wildlife, such as birds and small mammals (Côté et al. 2004), and modify nutrient cycling (e.g., Harrison and Bardgett 2004). However, vegetation changes slowly and can follow different trajectories, leading to uncertainty as to how ecosystems will eventually respond to deer management (Tanentzap et al. 2012). Nonlinear linkages between herbivore pressure and vegetation responses suggest that, in many instances, simply controlling deer may not return an ecosystem to its previous state (Tanentzap et al. 2009; Royo et al. 2010). Simulation modeling is one approach for overcoming the challenges in developing longer term management strategies by estimating future changes in vegetation and identifying the thresholds at which they occur (Kramer et al. 2003; Tremblay et al. 2004). Some models have recently included the effects of large mammalian herbivores such as deer (e.g., Seagle and Liang 2001; Weisberg et al. 2005), but none have yet formulated spatially explicit predictions. Furthermore, all of these models require relatively detailed parameterization because they monitor individual plants, and this limits their applicability at the landscape-level.

Extensive grazing by deer and sheep for many centuries, combined with other human land uses, is largely responsible for the image of a Scottish landscape of open moorlands (Holl and Smith 2007; Bennett 2009; but see Fenton 2008). Although much of Scotland would have been wooded historically (ca. 3000–5000 years ago; Tipping 1994; Smout 1997), less than 4% of land remains covered by native woodland (Mackenzie 1999). With increasing societal emphasis on the conservation of biodiversity (Gordon et al. 2004), and the economically unsustainable model of traditional sporting estates for recreational deer stalking (Wightman and Higgins 2000; MacMillan 2004), land management strategies in the Highlands are now reassessing the importance of native woodland (National Trust for Scotland [NTS] 2002; Featherstone 2004; Hobbs 2009). Conservation plans have been devised to both protect remaining fragments of native woodland and promote further regeneration, particularly within upland birchwoods, which are a priority habitat under the U.K. Biodiversity Action Plan (Forestry Commission Scotland [FCS] 2004). Attempts to increase birch woodlands are influenced by the management of herbivores (Kinnaird 1974; Miller et al. 1982, 1998; Pollock et al. 2005), especially populations of red deer (*Cervus elaphus*), which have increased across the Highlands over the last four decades (Clutton-Brock et al. 2004). Models of these woodlands can provide a powerful tool for land managers to balance the maintenance of traditional deer stalking with habitat conservation, but have not previously considered the impacts of herbivory (Manning et al. 2004; Towers et al. 2004; Hope et al. 2006).

Our objective was to predict the influence of browsing by red deer on landscape-level patterns of birch (primarily *Betula pubescens*) invasion. We achieved this by parameterizing a spatially explicit simulation model of upland birchwoods in the central Scottish Highlands; though our approach of using population-level data for young trees (<3 m tall) could easily be generalized to other systems where species are invading new habitats (e.g., Higgins and Richardson 1998; Sebert-Cuvillier et al. 2008; Travis et al. 2011) and toward simplifying existing individual-based simulation models (Picard et al. 2002). The model we developed simulates the growth, mortality, dispersal, and recruitment of birch in response to deer browsing and different ground cover. This then allows us to test how the expansion of upland birchwood varies with seed availability and ground cover along a gradient of deer browsing pressure, and whether it is influenced by the spatial configuration of seed sources. Our predictions occurred over a 30 year period, which is a timescale most relevant for our interests in birch regeneration and management. Conservation interventions are often planned to elicit responses within decades because they must demonstrate some return for investments to maintain financial and societal support (e.g., McCarthy and Possingham 2007; Dorrough et al. 2008). Moreover, by focusing on patterns of regeneration, and not long-term forest dynamics or succession, we simplify the need for detailed descriptions of how individual trees compete for light (Pacala et al. 1996). Rather, we show that patterns of regeneration across an afforested landscape can be replicated by modeling size-classes of young trees, in addition to the individual dynamics of larger isolated trees that weakly compete for light, as found across much of Scotland or in open vegetation types, such as savanna or grassland (e.g., Higgins and Richardson 1998). Overall, we define the underlying functional response of birch regeneration to landscape-level management.

## Methods

### Study site

Our study site was Creag Meagaidh National Nature Reserve, which occupies 3 940 ha within the central Scottish Highlands (56°57′N, 4°35′W). Mean annual precipitation is approximately 1 250 mm and the major soil type is humic podzols. Vegetation cover is primarily moorland dominated by heather (*Calluna vulgaris*) and purple moor grass (*Molinia caerulea*), with upland woodland occurring in small isolated patches and consisting of primarily downy birch (*B. pubescens*), and to a lesser degree silver birch (*B*. *pendula*), rowan (*Sorbus aucuparia*), and willow (*Salix* spp.). Red deer have been killed since 1986 in order to encourage woodland and scrub regeneration, reducing deer population densities from approximately 17.5 deer km^−2^ in 1986 to 1.7 deer km^−2^ by 2008, based on late winter population counts (Putman et al. 2005). Sheep (*Ovis aries*), roe deer (*Capreolus capreolus*), and sika deer (*Cervus nippon*) also occur in the region, but in very low numbers.

### Developing a model of birch invasion in the Scottish Highlands

**Figure 1:**
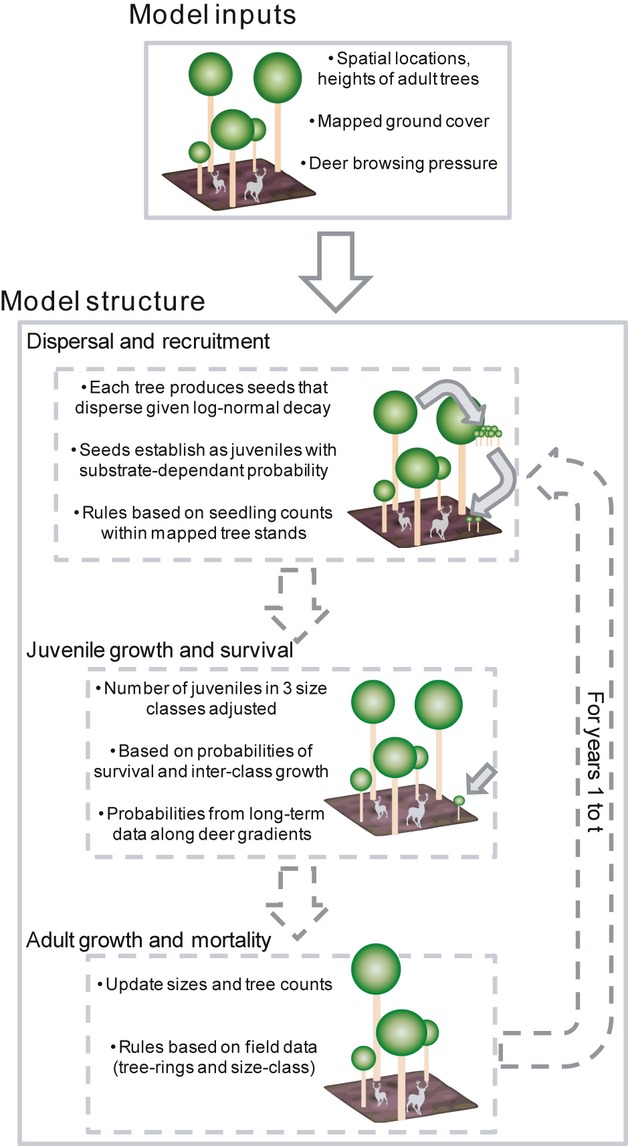
Outline of simulation model. Data used to parameterize each submodel denoted by hashed boxes. Inputs are fed to the model, which loops across the individual submodels for 1 to *t* years, after which the coordinates and heights of all trees in the landscape are output. We imposed two additional rules upon the model: (A) adult trees were also removed from the simulation when their crowns were 90% overtopped by neighbors; and (B) newly established juvenile trees could not progress to the taller height tier the following year.

Our approach for predicting birch population dynamics is entirely based on fitting mathematical functions at the levels of size-classes and individuals to field data collected at Craeg Mageidh (e.g., Pacala et al. 1996; Staver et al. 2009). The simulation model starts with a map of adult tree locations and makes spatially explicit predictions of how birch woodland spreads over time (Fig. 1). At each time step, the model uses the current distribution of adult trees (>3 m tall) to predict the number of seedlings recruited within every square meter of the landscape. Growth and survival of seedlings of different heights are then predicted by a series of probabilities that determine the proportion of seedlings surviving between years and moving from a small (0–2 m) to large (2–3 m) height class. These transition probabilities are ultimately influenced by deer browsing. Once seedlings grow beyond 3 m tall, they are classified as seed-bearing adults. Finally, the model grows and kills the adults. This routine is followed from the first to last year of the simulation (Fig. 1; see User Manual in Appendix S1 for description targeted for nonspecialists and conservation practitioners).

The simulation model was characterized by three “submodels”: juvenile recruitment, juvenile growth and mortality, and adult growth and mortality. Full details for data collection and parameter estimation are given in supporting information, so we summarize them here. First, we measured juvenile recruitment by surveying birch seedlings (<2 m height) and adult trees (≥3 m height) in six plots ranging from 0.6–4.0 ha. We recorded the height and location of each adult within a 300-m radius of the plots, and within 160–427 quadrats per plot (0.5 × 0.5 m in size), we counted the number of seedlings and visually estimated the ground cover of *Agrostis*-*Festuca* grassland, bog myrtle (*Myrica gale*), bracken fern (*Pteridium aquilinum*), heath (*Erica* spp.), heather, moss, purple moor grass, and *Vaccinium* spp. We assumed that seed dispersal from adults reached a peak within several meters and then declined exponentially. This was best described by a log-normal function and we fitted this function to our observations using maximum-likelihood methods (Greene et al. 2004). Model selection techniques showed that the log-normal model was more strongly supported than other functions, including those that allowed dispersal to be directionally dependent (Science Manual, Appendix S2). Our data also enabled us to estimate the potential number of recruits produced by each adult (*STR*) and favorability of ground cover for seedling establishment. We did so by simultaneously predicting the shape of the dispersal function, favorability of different ground cover, and value of *STR* that maximized the fit between predicted and observed seedling counts, given the observed spatial locations of adult trees. The different substrates also implicitly allow us to consider how establishment varies with different levels of understorey light and litterfall (e.g., *C. vulgaris* vs. *Vaccinium myrtillus*, Hester et al. 1991).

Second, we estimated juvenile growth and mortality using an 8 year dataset monitoring birch trees along six 1-km long transects spanning a gradient of 0–100% of trees being browsed. At 100 m intervals along each transect, a 100 × 2-m plot was located in which all trees emerging from unique seedlings were counted in 0–2, 2–3, and >3 m size-classes. The number of leader stems visibly damaged by mammalian herbivores was also recorded. We then estimated transition probabilities among the three height classes, and how these were affected by observed levels of browse damage, within a hierarchical Bayesian framework using Markov chain Monte Carlo sampling. This approach also allowed us to estimate the degree of interannual and spatial variation in growth and mortality. Matrix models such as ours have been successfully used to predict the effects of herbivores on woodland regeneration over medium-term timescales (e.g., 200 years, Staver et al. 2009), despite being criticized as inferior to approaches that incorporate individual-level variation, for example, integral projection models (IPMs, Ramula et al. 2009). We chose not to apply IPMs to our data as it would have required tenuously assuming how individual variation might be expressed among juvenile trees, and would have produced similar results to matrix models given that our dataset was suitably large (Ramula et al. 2009), and that growth in taller height tiers was neither size dependent nor autocorrelated (Pfister and Stevens 2003).

Third, we extracted increment cores from 40 adult birch trees at Corrour Estate, directly south of Craeg Mageidh. For each tree, we also recorded the diameter at breast height, standing height, and crown area. We calculated annual radial growth, averaged over the previous 8 years, and used this to predict adult height growth from standard allometric relationships (e.g., Russo et al. 2007). We then predicted the population size structure of all adults across our two sites using the observed growth rate but estimating density-independent mortality (Coomes et al. 2003). Adult mortality thus corresponded with the value that maximized the fit between the observed and the predicted size distributions. We did not simulate density-dependent mortality arising from competition for light as our interest was in tracking birch invasion rather than the dynamics of established stands. Density dependence is likely to also be weak for *Betula* as it regenerates poorly beneath canopy cover, often developing as nearly even-sized stands (Atkinson 1992; Mountford and Peterken 2000). However, we did remove adult trees from simulations when their crown area was 90% overtopped by neighbors. We also limited the number of individuals that could be recruited into 1 m × 1 m units of our landscape, whereby there was a maximum of 50 juveniles of any age <3 m tall in each 1 m^2^ area.

### Model validation

We validated our model by comparing predicted numbers of juvenile trees (<3 m tall) with observed counts in 2 × 100 m plots, accounting for the initial ground cover and annual variation in deer browsing in each plot. Counts were recorded in 10 plots located along each of five permanent transects in 2000. These data preceded measurements used to parameterize juvenile growth and mortality, so validation was independent of model parameterization. Starting conditions for validations were set by identifying all adult trees within 300 m of transects using digitized color aerial photographs from May 1990. We then parameterized our simulation model with adult tree distributions in the first year that a transect was measured, corresponding with 1988 for three transects and 1992 for the remaining two, and ran the model until 2000. The median count for each plot was calculated from 1000 simulations rather than the mean because distributions of predicted counts had very long right tails. We fitted a model to predict the median values from observed counts using a generalized linear model with Poisson error structure. The standard errors of the model parameter estimates were corrected by estimating a dispersion parameter to account for over dispersion (Ver Hoef and Boveng 2007). To test whether the slope and intercept of the model overlapped 1 and 0, respectively, as expected for an unbiased relationship between predicted and observed values, we calculated 95% confidence intervals (CIs). All models were fitted in *R* v2.14 (R Development Core Team 2011).

We also varied the most important predictors of juvenile tree densities: the potential number of recruits produced by each adult (*STR*) and survival in the 0–2 m height tier (*s*_1_), by 80–110% to try and minimize any bias in the predicted counts. We then selected the values of *STR* and *s*_1_ that produced a validation slope and intercept closest to 1 and 0, respectively, and used these in subsequent simulations. Our validation assumed: (1) ground cover varied little over time, so 2009 surveys could predict substrate favorability, (2) there was no competition between trees established along transects, if any, and incoming recruits, (3) soil seed banks were negligible (Miller and Cummins 2003), and (4) differences between predicted and observed values accumulate minimally over time (see Appendix S3 for further discussion).

### Simulating spread of birch woodland under different scenarios

#### Effects of seed availability, ground cover, and deer browsing

We tested the relative roles of seed availability, substrate favorability, and deer browsing in influencing birch invasion. First, we modeled the spread of *Betula* across a 2 × 2 km landscape over 30 years. Within the center of the simulated landscape, parent trees were randomly positioned within a 16 ha “core” block (hereafter “core invasion model”). We first fixed the starting density of adult trees at 250 or 500 trees ha^−1^, which is representative of a mature stand that can facilitate regeneration in open grassland and moorland (Cameron 1996; Mountford and Peterken 2000). Heights of parent trees were randomly drawn from a uniform distribution on the interval [3, 20]. We then allowed substrate favorability in 1 × 1 m grid cells to be drawn from a truncated normal distribution on the interval [0, 1], with *μ* varying from 0% to 100% in 10% intervals and *σ*^2^ representing the mean standard deviation (SD) in substrate favorability empirically observed across the permanent monitoring transects. Although this means that substrate favorability varies among years, it does not do so directionally, as might be expected where accumulating leaf litter drives a feedback among soil nutrients, ground cover, and eventually establishing trees (Miles 1981; Mitchell et al. 2007). Such processes can, however, be incorporated into our model, but were not included here because evidence suggests that they require at least 20–30 years to arise once a stand is established with several thousand trees ha^−1^ (Mitchell et al. 2007), and this is outside of the time frame of our simulations.

Simulations were run for each of the 11 substrate favorability levels, with the same randomly drawn set of parent trees, but the mean annual proportion of trees browsed by deer varied. Deer browsing was randomly drawn each year from a truncated normal distribution on the interval [0, 1], with *μ* varying from 0 to 1 in 0.1 intervals and *σ*^2^ = 0.13. The value of *σ*^2^ represented the mean SD in the proportion of damaged trees observed across the permanent monitoring transects from 2002 to 2010. At each substrate × browsing level, we performed 100 simulations and recorded the number of juvenile trees and total basal area (BA) of adults across our landscape after 30 years.

#### Effects of active seed source restoration

We repeated the simulations with a starting density of 500 trees ha^−1^ but altered the spatial arrangement of adults to test whether actively planting small, dense patches of adults better facilitates invasion (hereafter “patch invasion model”). Adult trees were subdivided into ten 0.1 ha patches within a 100-ha block that was positioned in the center of the 2 × 2 km landscape (e.g., Fig 1 in User Manual).

### Statistical analyses

We tested how birch regeneration was relatively influenced by deer browsing, substrate favorability, seed availability, and active management, corresponding with the planting of small patches of adults (“patch invasion model”). Our approach was to fit generalized linear models to predict juvenile tree densities and adult BAs from each level of simulations given our four aforementioned variables. We modeled juvenile tree counts in 0–2 and 2–3 m height classes across our entire landscape using a Poisson distribution with log-link function and accounting for over dispersion as in the validation. By contrast, adult BA was log-transformed and modeled over the same spatial extent using a Gaussian error structure. Active management was simply included as a binary predictor (0 = core invasion; 1 = patch invasion). We scaled our four explanatory variables to a mean of zero and SD of one so that their effects and 95% CIs would be directly comparable. All analyses were performed using *R*.

## Results

### Model development

The simulation model incorporated statistical fits that explained relatively large amounts of variation in field data (>54%; see Appendix S2 for details). First, we found that a log-normal function best explained dispersal of juvenile trees. The mean mode ± SD of the dispersal function occurred at 0.67 ± 0.08 m, and each tree dispersed 0.17 seeds m^−2^ over a 27 year period based on 154 felled juveniles. Seedling densities then declined exponentially until approaching zero seeds m^−1^ at a distance of >100 m from a parent tree (Table S1). Each adult produced, on average ± SD, a total of 1 046 ± 273 potential recruits. *Agrostis*-*Festuca* grassland was the best substrate for establishment, while the widely distributed *M. caerulea* was 85% less favorable (Table S1). Second, we found that seedlings had high survival once established; 95% credible intervals (CRs) in 0–2 m height tier using long-term measurements of juvenile size structure: 75–100% year^−1^. However, few of these juveniles transitioned to the taller height class (95% CRs: 0.1–1.2% year^−1^). Larger juveniles in the 2–3 m height tier survived at similar rates to those in the 0–2 m size-class, but transitioned at much higher probabilities (Table S1). Deer reduced survival and growth rates by a mean of 10% year^−1^, with the 95% CRs for this effect varying from 0% to 48% depending on browsing levels. Finally, the height growth of adult trees declined exponentially with diameter, whereby 3 and 25 m tall trees grew 0.019 and 0.005 m year^−1^, respectively. Mean adult mortality was 2.5% year^−1^ (95% CIs: 2.2–2.8% year^−1^).

### Validation

**Figure 2:**
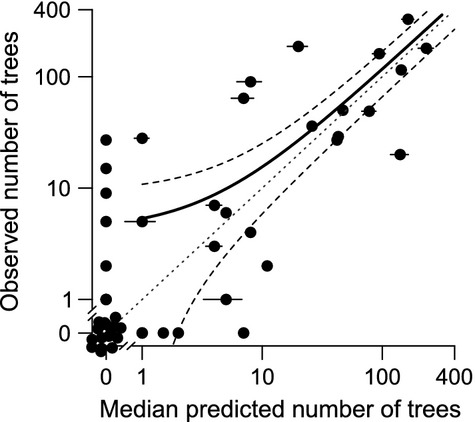
Model validation by comparing observed and predicted number of trees within fifty 2 × 100 m plots at Creag Meagaidh in 2000. Predicted values represent medians ± standard errors given 1000 simulations from adult tree locations in 1990. Dotted line denotes 1:1 relationship, while solid line represents fitted model between predicted and observed values ± 95% CIs. Equation of line: *y* = 4.21 + 1.13*x*; 22 plots with 0 trees observed and values were jittered for clarity.

Simulations initiated with 1990 tree maps generated slightly bias predictions of the numbers of juvenile trees 20 years later. The mean intercept was 4.2, which was relatively close to zero given the potential range of tree counts (95% CIs: −0.8 to 9.2), and the mean slope was 1.13 (95% CIs: 0.67–1.59). Predicted values thereby underestimated observed counts, but still explained a relatively large proportion of deviance (63%; Fig. 2). Varying the model parameters *STR* and *s*_1_ resulted in better fits between observed and predicted data, that is, mean slope equal to one (Table S2). We subsequently multiplied *STR* by 1.10 in our simulations and did not alter *s*_1_, because changing the latter did not improve the estimated intercept (Table S2). This ultimately increased the proportion of deviance explained by the validation to 66%.

### Model simulations

#### Effects of seed availability, ground cover, deer browsing, and active management

**Figure 3:**
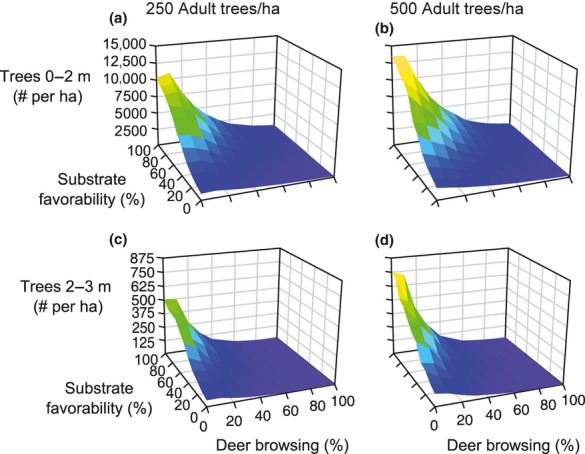
Numbers of juvenile trees in (A, B) 0–2 and (C, D) 2–3 m height tiers predicted after 30 years from initial densities of adult trees of either (A, C) 250 trees ha^−1^ or (B, D) 500 trees ha^−1^. Both substrate favorability and deer browsing varied in 10% intervals, and we performed 100 simulations at each combination of these two factors (*n* = 121). Plotted values represent means of 100 simulations; 95% CIs in Figures S1–S2.

**Figure 4:**
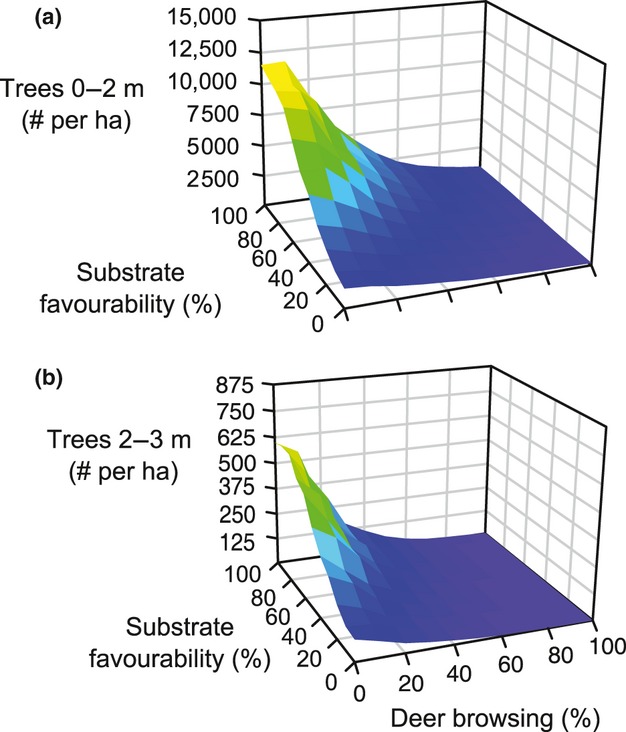
Numbers of juvenile trees in (A) 0–2 and (B) 2–3 m height tiers predicted after 30 years from 500 adult trees ha^−1^ initially located within ten 0.1 ha patches throughout the landscape, i.e. “patch invasion model.” Plotted values represent means of 100 simulations at each substrate favorability and deer browsing combination as in Figure 3; 95% CIs in Figure S3.

Both low deer browsing (≤20%) and high substrate favorability (≥60%) strongly increased the regeneration of juvenile birch trees (Figs. 3 and 4). However, juvenile abundances increased more steeply along the gradient of deer browsing than that of substrate favorability, especially when the latter was ≥80%. Greater initial densities of adults simply increased the magnitude of regeneration, but the overall shape of responses was similar at 250 and 500 trees ha^−1^, and independent of the spatial arrangement of adults, i.e. between the “core” and “patch” models. As expected, regeneration was greatest at 0% deer browsing and 100% substrate favorability, and there was some overlap among the 95% CIs for the simulations at 0% and 10% browsing and 90% and 100% substrate favorability (Figs. S1–S3). There was large overlap in predicted regeneration across the entire range of potential browsing rates where substrate favorability was ≤20% (Figs. S1–S3).

The BA of adult trees varied little across the simulations within a fixed starting tree density, likely because insufficient time elapsed for differences to be expressed (Figs. S1–S3). The simulations started with no juveniles in the landscape, as is common across much of the Highlands, so as parent trees died there was a time lag before new trees recruited. Consequently, BA of the landscape was ca. 17–38% lower than at the start of simulations after 30 years, but this was exceeded when simulations were run longer, for example, 200 years.

#### Relative importance of deer, ground cover, seed availability, and active management

Deer browsing had the greatest absolute effects on regeneration in our statistical models of simulation predictions (Table 1). The functional relationship between regeneration and deer browsing followed a negative exponential shape because juvenile tree counts were modeled on a log-scale (Fig. S4). Thus, juveniles accumulated most rapidly where no trees were browsed, and this rate of regeneration declined exponentially with browsing; the rate of change (i.e., first-order derivative) of an exponential function is equal to the function itself. The negative effects of deer were also greater in the 2–3 m than 0–2 m height tier (Table 1), because browsing prevented tree growth more than establishment and survival (see Section 3, Appendix S2).

**Table 1:** Mean effects (95% CIs) of four variables on densities of trees in 0–2 and 2–3 m height tiers, and BA (m^2^) of adult trees >3 m tall, predicted after 30 years from model simulations

Variables	Tree density (0–2 m)	Tree density (2–3 m)	Basal area
Deer browsing	−0.87 (−0.87 – −0.86)	−1.47 (−1.49 – −1.46)	−6.90 (−7.24 – −6.56)
Substrate favorability	0.59 (0.59 – 0.60)	0.55 (0.54 – 0.56)	5.86 (5.52 – 6.20)
Initial adult tree densities	0.13 (0.13 – 0.14)	0.15 (0.14 – 0.16)	380.0 (380.0 – 381.0)
Active management scenario (“patch invasion model”)	−0.06 (−0.06 – −0.05)	−0.06 (−0.07 – −0.05)	−1.55 (−1.94 – −1.16)
Proportion of deviance explained by full model	0.85	0.73	0.99

Both substrate favorability and deer browsing varied in 10% intervals, and we performed 100 simulations at each combination of these two factors (total *n* = 12,100). Variables were all standardized to a common scale, so their effects are directly comparable and “significant,” that is, 95% CIs do not overlap zero.

Substrate favorability had the second-strongest relative effect on regeneration, followed by the initial densities of adult trees (i.e., seed availability). Both favorable substrates and high seed availability increased regeneration, and the former could offset ca. 68% and 37% of the effect of browsing in the 0–2 and 2–3 m height tier, respectively (Table 1; Fig. S4). Our active management scenario, corresponding with the planting of small, dense patches of adult trees across the landscape (=patch invasion model), had no benefits for facilitating regeneration (Table 1).

Deer, ground cover, and active management negligibly affected BA across simulations, as compared with the effect of initial adult densities (Table 1). As explained above, this finding reflected the temporal scale of our model, suggesting that any management actions are unlikely to increase BA in <30 years.

## Discussion

We found that high stocking of red deer reduces the regeneration and expansion of Birchwoods in the central Scottish Highlands, as known for at least 100 years (Crampton 1911; Darling 1947; Miller et al. 1982, 1998), but we have now explicitly defined how managers can alter multiple landscape features to recover tree densities. Ultimately, management actions mitigating the negative impacts of browsing require suitable ground cover for seedling establishment. Given favorable ground cover, defined by ≥60% probability of seedling establishment, birch regeneration occurs where deer browse ≤10% of trees.

Our study provides a powerful tool for land managers to reverse the decline of a habitat prioritized for conservation action (Forestry Commission Scotland [FCS] 2004). The need for quantitative tools to support conservation management decisions is well recognized, but the difficulty in developing these has hampered their broader application (Tremblay et al. 2004). Managers are instead faced with the challenge of often making decisions based upon a qualitative and imperfect understanding of how systems operate (Tremblay et al. 2004). Here, we show how the simplification of key ecological processes can better inform management by making simulation models more analytically tractable. For example, data from size-classes rather than individual trees still manage to replicate observed patterns of tree regeneration. Although modeling individuals is certainly desirable, the necessary data are difficult to obtain for long-lived organisms, such as trees. Small trees occur in such high numbers that population-based models can be parameterized accurately, particularly at a landscape scale, and combined with approaches for individually tracking large trees, which are more important for monitoring ecosystem properties such as BA (Picard et al. 2002). While other parameters are undoubtedly important, such as biogeochemical feedbacks and resource availability (Mitchell et al. 2007), these can also be approximated by indicators that are easier to measure, such as our ground cover classes, which are a direct outcome of these different processes.

The goal of our model was to predict juvenile tree densities and it did so with accuracy, despite criticisms that may be directed toward some of our model parameterization, such as the assumptions about adult mortality. Our validation showed that predicted tree counts, which represented outputs from a dynamic simulation model, closely matched observed values. No other spatially explicit, numerical, simulation models have validated the impacts of deer herbivory over such broad landscape scales as studied here. By quantifying the uncertainty underlying model predictions, our simulation model has much broader potential for informing managers of the actions needed to restore and expand birch woodlands.

### Management strategies for maximizing birch invasion

Our results now enable managers to quantify the levels of three landscape features that are required to maximize birch invasion: red deer populations, ground cover composition, and the availability of adult seed sources.

#### Deer

Our finding that regeneration is strongly dependent on browsing damage, and that the latter can only be achieved through very low deer densities (see Section 5, Appendix S2), emphasizes the need for managers to address the impacts of deer rather than their densities. Others have similarly recommended that managers focus on how features of interest, such as biodiversity or commercial resources, respond to herbivores rather than animal densities per se (Gordon et al. 2004; Morellet et al. 2007; Putman et al. 2011). More generally, the goal of management should be to manipulate densities of deer such that their impacts on conservation objectives are deemed tolerable (Parkes 1993). Existing policy frameworks for managing wild deer in Scotland are primarily based upon monitoring habitat condition rather than deer population size (Deer Commission for Scotland [DCS] 2008), so our model is immediately applicable in this context. Simply relating deer density to browsing impacts is problematic because it ignores the fact that the effects of deer on vegetation communities are nonlinear (Gill and Morgan 2010; Tanentzap et al. 2012), and vary with factors such as population age and sex structure, season, and hunting pressure (Miller et al. 1982; Clutton-Brock and Albon 1989; Bee et al. 2010).

Several means exist for manipulating deer population size at large spatial scales. The primary approach is through culling animals, as implemented at our study site (Putman et al. 2005), and we recommend this method given our results demonstrating the need for large-scale reductions in browsing to stimulate birch regeneration. Other control methods, such as relocation or sterilization, are inappropriate at the landscape scale. Conservationists have also recently raised the possibility of reintroducing wolves (*Canis lupus*) into the Highlands to regulate deer population dynamics (Manning et al. 2009). In the western USA, similar reintroduction of large carnivores has increased the height and density of herbivore-preferred plants across landscapes (Beschta and Ripple 2009). While the restoration of such “natural” processes is appealing (Manning et al. 2009), it would require fencing large estates (i.e., tens of km^2^) to keep animals from migrating outwards, and the associated costs may prove prohibitive.

#### Ground cover

We found that birch regeneration depends on favorable ground cover for seedling establishment, such as *Agrostis*-*Festuca* grassland and *C. vulgaris* (Table S1). Birch is strongly intolerant of shade, so establishes poorly beneath closed canopies (Atkinson 1992), such as found beneath dense swards of *M. caerulea*, a dominant community type in the central Highlands (Hester et al. 1996). Although *C. vulgaris* can also shade birch seedlings (Atkinson 1992), light penetration was much higher than beneath *Molinia* at our study site, thereby explaining its greater favorability. Khoon and Gimingham (1984) did find that birch recruited beneath *C*. *vulgaris*, as long as stands were immature or in a canopy-breakup stage.

We suggest that selective disturbances can help managers create favorable substrate for birch regeneration after deer culling, especially where recalcitrant vegetation persists (Miller et al. 1998). For example, grazing and trampling by livestock increase light penetration to the soil surface, potentially favoring seedling establishment, without entirely inhibiting regeneration (Pollock et al. 2005). Reintroduction of wild boar (*Sus scrofa*) into the Highlands may also be desirable, and would complement the effects of livestock because animals root in vegetation dominated by bracken (*P. aquilinum*) during winter (Sandom et al. 2013). Both the promotion of extensive livestock production and regulated reintroduction of boar may confer additional socioeconomic benefits for local communities by enhancing important components of the Highland economy, that is, farming and sport shooting (Wightman and Higgins 2000). Prescribed burning can also help managers create favorable substrates by increasing light availability and favoring site colonization through seed dispersal, for which birch is adept (Atkinson 1992), but will need to consider carefully the responses of surrounding vegetation.

#### Adult seed sources

We found that the availability of adult seed sources influences regeneration, though to a much lesser extent than deer browsing and ground cover. As seed sources are becoming rarer across the Highlands, due to decades of limited canopy tree regeneration (Kinnaird 1974; Miller et al. 1998; Pollock et al. 2005), managers may artificially wish to increase seed rain onto the landscape. One option may be to fence any available regeneration to allow trees to escape the reach of browsing deer and eventually mature. However, the complete removal of grazing animals is undesirable because it can promote the accumulation of recalcitrant ground cover (Miller et al. 1998). Fences also detrimentally affect grouse (*Lagopus* spp.) and other native birds (Baines and Summers 1997), are difficult to maintain in remote mountainous areas, and represent barriers to human access in a culture that promotes universal access to the land. Another option may be to sow seeds artificially, but current techniques likely cannot achieve landscape-level regeneration (Willoughby et al. 2007).

#### Other management

We specifically investigated how the creation of small wooded patches would accelerate regeneration. Benayas et al. (2008) theorized that this would expedite restoration in low productivity, agricultural landscapes. However, we found that ten 0.1 ha patches of woodland actually reduced regeneration relative to afforestation from a larger 16 ha contiguous patch. In smaller, fragmented populations, individuals are at greater risk of demographic stochasticity, and so patches can more easily go extinct (Benayas et al. 2008), likely explaining our findings. While this is an outcome of the size of our simulated patches of adult trees, we deemed these to be a realistic scenario for active management in this region. Importantly, our simulation model now allows testing of specific restoration scenarios to inform conservation actions.

#### Monitoring outcomes

Managers also need approaches for tracking the responses of regeneration to changes in ground cover, deer browsing, and seed availability. We suggest that simply counting trees annually along permanent monitoring transects is useful. Across larger landscapes, automated tree counts may be derived from remote sensing technologies (Dralle and Rudemo 1996; McCombs et al. 2003). In addition to regeneration, managers must monitor the responses that they directly manipulate in order to inform future decision making. Browse damage should therefore be tracked along permanent monitoring transects as it is the most important determinant of regeneration. At the landscape scale, other indices may be easier to measure and can be calibrated against local browse damage. For example, the body mass of fawns tracks temporal variation in browsing, because it declines with population size, and thus deer impacts, and can be easily collected where culling occurs (Morellet et al. 2007).

### Conservation implications

Our study also has implications for other conservation initiatives. First, governments in the UK have committed to reducing carbon (C) emissions by 2020, such as through woodland conservation (Committee on Climate Change [CCC] 2008). Our model can easily generate future estimates of C stocks in aboveground biomass in response to deer management and/or restoration of adult seed sources. For example, published allometric equations between tree diameter and biomass suggest that an 8.5 m tall birch contains approximately 10.4 kg C (Bunce 1968). Establishing at least 5800 trees taller than this height would thereby increase C by at least 60 *t* C after 30 years, as we found, on average, where a mean of 10% of trees were browsed in a landscape with 80% substrate favorability and an initial population of 8000 adults. Of course, this does not consider how regeneration might alter soil C storage, particularly in peatlands. Second, our model can be used to predict outcomes for other threatened taxa targeted by conservation interventions. For example, black grouse (*Tetrao tetrix*) is one of the most rapidly declining bird species in the U.K. (Forestry Commission Scotland [FCS] 2008). Grouse in southern Norway select winter habitats with high densities of birch >8 m tall (Hjeljord et al. 1995), so knowledge of birch regeneration patterns can be input into predictive models of grouse distributions. Overall, our model provides a much needed tool for land managers to predict better how wildlife management affects biodiversity outcomes, especially given the relatively long-time scales on which vegetation responds to changes in herbivore densities and the nonlinearities that are involved.

## Supporting Information

**Appendix S1.** User manual.

**Appendix S2.** Science manual.

**Appendix S3.** Additional methods.

**Appendix S4**. Model video (*.wmv).

**Appendix S5**. Original juvenile transect data (*.txt).

**Appendix S6**. Modified juvenile transect data (*.txt).

**Table S1.** Submodel parameter estimates.

**Table S2.** Model tuning.

**Figure S1.** CIs for core invasion model at 500 adults ha^−1^.

**Figure S2.** CIs for core invasion model at 250 adults ha^−1^.

**Figure S3.** CIs for patch invasion model.

**Figure S4.** Regeneration along deer and substrate gradients.
