# Supplementary material for: Getting the biggest birch for the bang: restoring and expanding upland birchwoods in the Scottish Highlands by managing red deer
Source: Ecol Evol. 2013 May 22;3(7):1890–901. doi: 10.1002/ece3.548 (PMC3728932; doi:10.1002/ece3.548)
Supplement: Supplementary file 1 [file ece30003-1890-SD1.pdf]

Birch Invasion Model – User Manual

Andrew J. Tanentzap, James Zou & David A. Coomes

February 3, 2013

Contents

1 Overview **1**

2 Model inputs **2**

3 Model runs **3**

3.1 Adult growth and mortality 4

3.2 Juvenile growth and mortality 5

3.3 Juvenile recruitment 6

4 Model outputs **7**

4.1 Tree data 7

4.2 Landscape visualization 7

1 Overview

The purpose of this document is to describe how the Tanentzap, Zou & Coomes birch invasion model performs simulations. We have divided this manual into three sections: 1) model inputs; 2) functionality of the models different routines; and 3) model outputs. The three sections respectively correspond with the operation of the model at $t = 0, 0 < t < N$, and $t = N$, where t is an annual time step and N is the total length of the simulation period. Details of the underlying functions of

the model, and their parametrization, are described in the accompanying Science Manual.

The birch invasion model is implemented within the `R` programming language (R Development Core Team 2011) and can operate on any platform that has installed the `R` software environment (available for free at: `http://cran.r-project.org`). A visualization option is also available and can be turned on or off by changing a `TRUE` / `FALSE` statement in the head of the model code. Source code required to run the model is available upon request from A. J. Tanentzap: ajt65@cantab.net.

Simulations can take several days to run, so patience may be necessary on older computers! For example, a simulation of 30 years with the "core invasion" model, initialized with 500 trees ha^{-1}, a mean of 10% of trees browsed, and 80% mean substrate favourability takes approximately 11 hours using the 64-bit `R v2.14.1` terminal in Windows 7 on a Intel® Core™ `i7-2600` processor (`8MB Cache, 3.4GHz`) with 16`GB` Dual Channel DDR3 SDRAM at `1333MHz - 4 DIMMs`.

2 Model inputs

The model makes use of four primary inputs: number of simulation years, initial number of adult trees per ha (where adults are trees ≥3 m tall), mean favourability of substrate cover, and the mean probability of trees being browsed by deer. Both the mean favourability of substrate cover and the probability of trees being browsed by deer can be specified either as single values or a vector of values for each year of the simulation. An important distinction is that although these values are 'specified', they in fact represent mean estimates and so may differ from estimates subsequently used in model calculations. Simulations in the Tanentzap, Zou & Coomes manuscript drew values of substrate favourability from a truncated normal distributions, with the user specified value respresenting the mean and the standard deviation based upon observed values. By contrast, the mean annual probability of browsing was added to estimates of annual and spatial variation in the effects of deer to estimate a 'true' probability of browsing in each year.

Following specification of model parameters, a 2 km × 2 km landscape is constructed and populated with adult trees. Trees are randomly positioned within either a 16 ha region ('core invasion' option) or equally spread across 10 0.1 ha patches within a larger 100 ha region ('patch invasion' option) positioned in the centre of the landscape (Fig. 1). For each tree, a height is randomly drawn from

a uniform distribution on the interval [3, 25] and each tree is assigned an 'age', corresponding to the year the tree entered the model. Substrate favourability is added to the landscape within 1 m × 1 m grid cells.

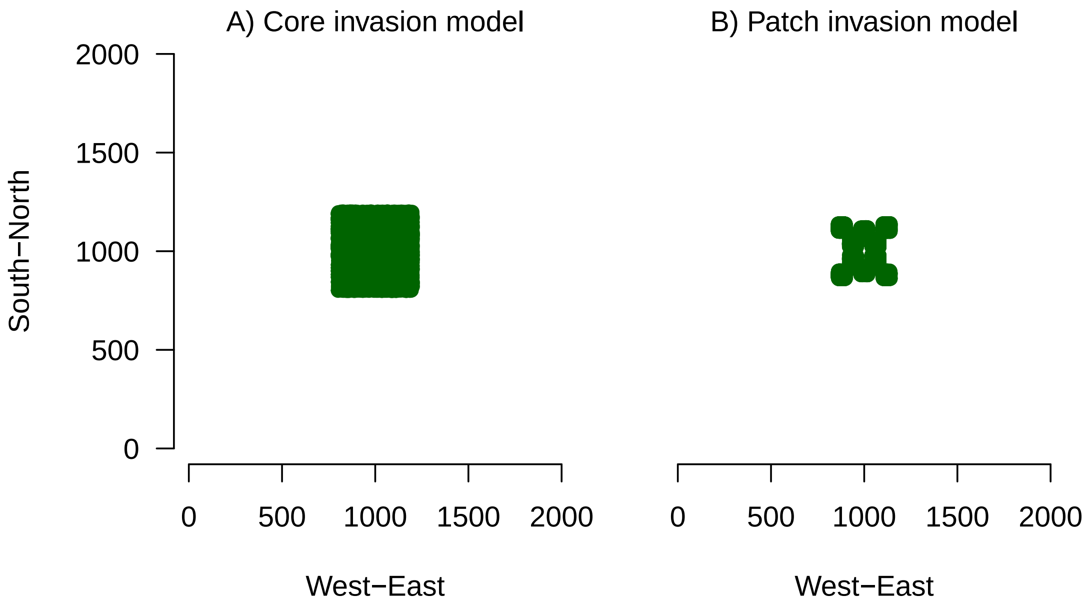

Figure 1. Examples of A) core invasion and B) patch invasion models viewed in two-dimensions. Each point corresponds with a tree, with 500 trees ha^{-1} spread across a A) 16 ha patch and B) within ten 0.1 ha patches within a 100 ha patch positioned in the centre of the simulated landscape.

Input features can be customized by modifying the source code. Thus, the model allows for simulation of birch invasion from existing stands elsewhere in the central and western Scottish Highlands. Customization options include the sizes of the landscape and regions in which adult trees are positioned. A vector of known coordinates for trees can also be supplied to the model. Additionally, it is possible to alter assumptions regarding the distributions of substrate favourability and whether substrate favourability and deer browsing vary annually.

3 Model runs

The model uses a 'loop' routine across the number of specified years after initial parameters are set and the landscape is constructed and populated with trees. In other words, the operations outlined in the following subsections are repeated for N number of years in the order in which they appear.

3.1 Adult growth and mortality

Adult trees are grown and killed during each time step. Growth occurs simply by applying the height growth function described in the Science Manual to each individual ≥3 m tall. By contrast, mortality arises due to two processes: i) the empirically estimated mortality rate (M) of the population (which can be customized); and ii) crown overlap, which is intended to capture asymmetric competition for light that reduces growth and consequently increases mortality (Weiner 1990; Kobe *et al.* 1995; Adler 1996). M is applied as a stochastic behaviour, such that a random number R between 0 and 100 is generated for each adult tree, and trees are identified for removal where $R \leq M$. The model then identifies trees that have 90% of their canopy overtopped by neighbouring individuals. The overtopping routine first calculates the distance of each tree to taller trees in the landscape and this measure is used along with crown radius to determine which trees overlap. If a focal tree has only one neighbour, the area of its crown that is overtopped (A) is calculated as:

$$\begin{aligned} A &= \pi r_0^2 \cos -1 \left[\frac{d^2 + r_0^2 - r_1^2}{2dr_0} \right] + r_1^2 \cos^{-1} \left[\frac{d^2 + r_1^2 - r_0^2}{2dr_1} \right] \\ &- \frac{1}{2} \sqrt{(-d + r_0 + r_1)(d + r_o - r_1)(d + r_0 - r1)(d - r_0 + r_1)}, \end{aligned} \tag{1}$$

where r_0 is the radius of the focal crown, r_1 is the radius of the taller neighbouring crown, and d is the distance between the two trees. If a tree has more than one neighbour, the model then tests whether any of these trees entirely overtop a focal tree.

We implement a Monte Carlo approach when focal trees are not entirely overtopped by one of two or more neighbours. Calculation of the overlap of more than two circles (i.e. crowns) is analytically complicated, particularly from the perspective of deriving closed-form algebraic expressions (Fewell 2006). The case of how much a focal circle is overlapped by more than three neighbours is especially complex and does not have a simple solution that can be implemented in our model. Our approach is to first superimpose a mesh upon the crown of each neighbouring tree in 0.25 m intervals. If this approach generates >100 unique points, points are randomly sampled 100 times without replacement. We then count the number of points from neighbouring trees that occur within the crown of the focal tree and divide this number by the total number of tested points to generate the proportion

of area that a focal crown is overlapped. Increasing the resolution of the grid or the number of points for testing is a major bottleneck of the model, particularly over large areas and with many trees to test.

Trees with ≥90% of their crown overtopped by neighbours are identified and removed from the model at the end of the 'Adult mortality' routine along with those trees that are identified as being dead simply due to annual mortality (i.e. M).

3.2 Juvenile growth and mortality

Juvenile trees are grown and killed each time step within discrete height classes (0 – 2 and 2 – 3 m). The probability of a juvenile either entering a taller height class or dying is constant within a height class and random variation due to year is added to each mean estimate:

$$y_{ijt} = \mu_{ij} + \tau_t, \tag{2}$$

where y_{ij} represents demographic rater i (either the proportion of trees that grow or survive, $y_{1,t}$ and $y_{2,t}$, respectively) within height tier j at time t, and τ_t is random variation due to year and is $\sim N(0, \epsilon_\tau)$. By drawing parameters from probability distributions for each iteration of the simulations, we can better capture variability in demographic rates than if parameters were constant estimates. Values estimated from the matrix model described in the Science Manual replace μ_{ij}, and ϵ_τ with known quantities.

$N_{jt}y_{1j}$ trees in each height tier j are randomly sampled to survive at each time step t, where N_{jt} is the total number of trees in a height tier j. From the survivors, individuals are moved to the next height tier with a probability of y_{2j}, i.e. $N_{jt}y_{1j}y_{2j}$ trees advance to the next height tier. Juvenile trees in the 2 – 3 m height tier that are selected to advance up a height tier become 'adults' and are assigned heights by randomly sampling from a distribution that is uniform on the interval between 3 and 4. Year-old seedlings are prevented from growing out of the first height tier the following year to avoid unrealistic growth spurts. Deer affect (Eq. 2) by multiplying growth and survival probabilities in the 0 – 2 m height tier by $2 - 2\text{logit}^{-1}(dp)$, where p is the probability of a tree being browsed in the landscape and d is the effect of browsing on growth and survival (see Science Manual for details).

3.3 Juvenile recruitment

Juveniles are added to the landscape using the isotropic lognormal dispersal kernel described in the Science Manual. The first step is to calculate the number of seedlings produced by each adult, and each of these seedlings is then assigned a dispersal distance and direction. Seedling production is standardized to an annual interval by multiplying the total number of seedlings produced by a normalizing function that incorporates annual survival and growth of juveniles in the 0 – 2 m height tier and the effect of deer (see Sections 3.1 – 3.2 in the Science Manual for detailed explanation). Dispersal distance is determined by randomly sampling once for each new juvenile from a lognormal distribution (parameters defined as in the Science Manual), and direction in radians is determined by randomly sampling once for each new juvenile from a distribution that is uniform between $-\pi$ and π. Coordinates of each seedling are rounded down to the nearest whole number in order to be associated with a grid cell with a given substrate favourability. The total number of seedlings in each 1 m × 1 m grid cell is then multiplied by the substrate favourability of the cell. Although this approach can assign the same coordinates to multiple individuals, the probability that a juvenile will grow into an adult is so small (i.e. $<1 \times 10^{-7}$, assuming individuals survive) that juveniles will not overlap as adult trees. Model customization that alters the growth and survival probabilities of juveniles can however alter the source code to retain the original coordinates of each juvenile (i.e. unrounded values).

The total number of juveniles in a 1 m × 1 m grid cell is also limited to 50 to prevent unrealistically large numbers of individuals from accumulating, i.e. thousands per 1m^2. If the number of seedlings that are recruited into a 1 m × 1 m at a given time step exceeds 50, the total number of juveniles in that cell is calculated, and the total number of recruited seedlings is truncated to the difference between 50 and the number of existing juveniles. Consequently, zero new seedlings can be added where 50 juveniles are already present within a 1 m × 1 m grid cell.

4 Model outputs

4.1 Tree data

The model can technically return any information described in the User Manual. Presently, a `*.csv` file (comma-separated values) is written to a user specified directory at the end of the simulation. The `*.csv` file contains four columns corresponding to the x and y coordinates of each tree, along with its height and model age. Trees that are deposited onto the landscape during the final simulation year are not included in the model output.

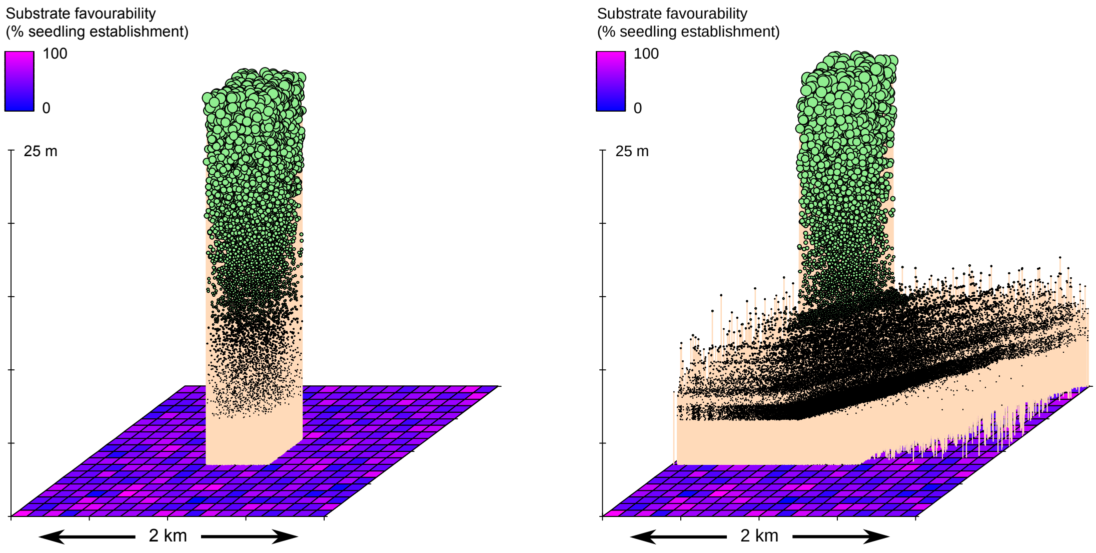

Figure 2. Example of model output plot generated from "core invasion" option after 30 years. Starting adult tree density was 500 trees ha^{-1}, and a mean of 10% of trees were annually browsed by deer. Left-side is start of simulation at time = 1, and right-side is time = 30.

4.2 Landscape visualization

A 3D landscape visualization option is included in the model that utilizes the `scatterplot3d` package (Ligges and Mächler 2003). The visualization can plot the simulation landscape at any time step, with the z-axis of the plot corresponding to tree height (set to a maximum of 25 m; Fig. 2). Tree trunks and crowns

are added to the plot and scaled relative to tree height. Ground cover is also plotted but within 10 m × 10 m grid cells to minimize computational cost. The ground cover is plotted using a base colour of purple (to mimic flowering *Calluna vulgaris*) and the intensity of colouring is scaled to correspond with actual values of substrate favourability, where the gradient in substrate favourability is reported through the addition of a legend. Future customization of the plot can include altering its background colour to gray in order to replicate sky conditions in the Scottish Highlands.

References

Adler, F. R. 1996. A model of self-thinning through local competition. *Proceedings of the National Academy of Sciences* **93**:9980–9984.

Fewell, M. P. 2006. *Area of common overlap of three circles.* DSTO Defence Science and Technology Organization, Edinburgh, Australia.

Kobe, R. K., S. W. Pacala, J. A. Silander Jr, and C. D. Canham. 1995. Juvenile tree survivorship as a component of shade tolerance. *Ecological Applications* **5**:517–532.

Ligges, U., and M. Mächler. 2003. Scatterplot3d - an R package for visualizing multivariate data. *Journal of Statistical Software* **8**:1–20.

R Development Core Team. 2011. *R: A Language and Environment for Statistical Computing.* R Foundation for Statistical Computing, Vienna.

Weiner, J. 1990. Asymmetric competition in plant populations. *Trends in Ecology & Evolution* **5**:360–364.
