# Supplementary material for: Getting the biggest birch for the bang: restoring and expanding upland birchwoods in the Scottish Highlands by managing red deer
Source: Ecol Evol. 2013 May 22;3(7):1890–901. doi: 10.1002/ece3.548 (PMC3728932; doi:10.1002/ece3.548)
Supplement: Supplementary file 2 [file ece30003-1890-SD2.pdf]

Birch Invasion Model – Science Manual

Andrew J. Tanentzap, James Zou & David A. Coomes

February 10, 2013

Contents

1 Introduction **3**

2 Juvenile dispersal and recruitment **3**

2.1 Spatial seedling dispersal behaviours 3
2.2 Model estimation and selection 5
2.3 Results of seedling dispersal models 7

3 Juvenile growth and survival **8**

3.1 Derivation of size-based matrix model 8
3.2 Incorporation of deer browsing into matrix models 10
3.3 Dataset preparation 12
3.4 Derivation of model likelihood function 14
3.5 Parameter estimation 14
3.6 Model assessment 16
3.7 Conversion of parameter estimates to annual rates 17
3.8 Results of juvenile growth and survival models 17

4 Adult growth and mortality **20**

4.1 Adult growth 20
4.2 Adult mortality 21
4.3 Competition for light and density-dependent mortality in adults . . 23
4.4 Results of adult growth and mortality models 23

5 Measures of deer browse pressure **25**
5.1 Indicators of deer browse pressure 25
5.2 Models relating deer damage to animal density 27
5.3 Relationships between deer browsing and density 28

6 References **30**

7 Appendix S1 – JAGS code for matrix model **35**

1 Introduction

The purpose of this document is to detail the mathematical and statistical parameterization of the three submodels of the Tanentzap, Zou & Coomes birch invasion model: 1) juvenile dispersal and recruitment; 2) juvenile growth and survival; and 3) adult growth and mortality; a section of text is devoted to each submodel. A fourth section describes the relationships between deer browse pressure and deer population counts at our study site, Creag Meagaidh National Nature Reserve, Scotland. Within each section, we use separate subsections to report the methods and results pertaining to the parametrization of each submodel. Operation of the model is described in the accompanying User Manual.

2 Juvenile dispersal and recruitment

2.1 Spatial seedling dispersal behaviours

We used mapped stands of adult and juvenile trees at Creag Meagaidh to predict juvenile dispersal using a deterministic model. We predicted the potential number of seedlings produced by a tree as a function of tree height (Z, m; Ribbens *et al.* 1994):

$$g(Z) = \mathrm{STR}\left(\frac{Z}{8.5}\right)^{\beta}, \tag{1}$$

where STR and β are estimated parameters that scale seedling production to parent tree size and were relative to a 8.5 m tall tree, which was the mean across adults. The minimum size for reproduction was set at 3 m (i.e. our definition for an 'adult' tree). The distribution of seedlings in relation to parent trees measured in seedling plots was then fit to isotropic and anisotropic dispersal kernels employing both Weibull (f_1) and lognormal (f_2) distributions (Greene *et al.*, 2004). The isotropic form assumes that dispersal is equally likely at the same distance in all directions from a parent tree and the Weibull and lognormal kernels were of the following forms (Eqs 2 and 3, respectively; Ribbens *et al.* 1994; Clark *et al.* 1998; LePage *et al.* 2000; Greene *et al.* 2004):

$$f_1(d_{ij}) = \frac{1}{\eta}e^{\phi d_{ij}^{\gamma}}, \tag{2}$$

$$f_2(d_{ij}) = \frac{1}{\eta} e^{-\frac{1}{2}\left(\frac{\ln(d_{ij}/X_0)}{X_b}\right)^2}, \tag{3}$$

where d_{ij} is the distance from point i to the parent tree j, ϕ and γ are estimated parameters, X_0 and X_b are the estimated mode and steepness of the lognormal kernel, and η is a normalisation constant equivalent to the arcwise integration of the dispersal kernel. The Weibull model differs biologically from the lognormal by assuming that seedling density is greatest at the base of parent trees. This may arise, for example, if seeds are large and frequently collide with parent trees such that their vertical acceleration and/or horizontal speeds are reduced, leading to less distant dispersal (Greene *et al.*, 2004; Pounden *et al.*, 2008). Similarly, maximum seedling densities at the base of trees may be identified in inverse modelling for species of seeds dispersed by avian granivores that roost in trees of the same species from which they have recently ingested seed (Schupp *et al.*, 2002). Combining Eq. 1 with either Eq. 2 or Eq. 3 (i.e. f_n) eventually allows us to predict the number of seedlings (R_i) per m^2 at a given point i:

$$R_i = \mathrm{STR} \sum_{j=1}^{n} \left(\frac{Z_j}{8.5}\right)^{\beta} f_n(d_{ij}), \tag{4}$$

In contrast, anisotropic kernels assume that dispersal distance depends on the direction in which seeds are released from a parent tree because of the directional effects of prevailing winds. Anisotropic and isotropic kernels are of the same general form, except that the dispersal parameter ϕ of the Weibull kernel and the mode of the lognormal kernel X_0 are modified (Eqs 5 and 6, Gómez-Aparicio and Canham 2008):

$$X_0'(d) = X_o - [A \times \cos(\theta - \delta)], \tag{5}$$

$$\phi' = \phi - [A \times \cos(\theta - \delta)], \tag{6}$$

where A is the amplitude of the anisotropic effect, θ is the angle of the maximum dispersal distance, and δ is the angle from each parent tree j to point i. A also becomes incorporated into the normalization constant as $\eta = \eta(A, X_0, X_b)$.

Seedling densities depend on the establishment success of propagules in addition to the number of propagules that arrive at a given point (R_i). We predicted the proportion of seeds that establish in each substrate type (g_k) as follows (LePage

et al., 2000):

$$F_i = \sum_{k=1}^{8} g_k c_{ik}, \tag{7}$$

where $c_i k$ is the proportion of area (0.25 m^2) at point i that is covered by substrate type k, and F_i is the weighted substrate favorability for each point i. F_i values scaled relative to each other by dividing each value by the largest estimated F_i. Both the isotropic and anisotropic kernels (Eq. 4) were multiplied by Eq. 7. Although we did not measure the influence of light on seedling establishment (Caspersen and Saprunoff, 2005), the incorporation of different substrates implicitly considers establishment under different levels of understorey light (e.g. *Calluna vulgaris* versus *Vaccinium myrtillus*, Hester *et al.* 1991; Millett *et al.* 2006).

2.2 Model estimation and selection

We used maximum-likelihood methods to estimate parameters for models fit to our seedling census (see main text). The negative log-likelihood of each model was minimized with a Poisson error structure using 100 000 iterations of the simulated annealing algorithm for the `optim` function in `R` (R Development Core Team, 2011). Simulated annealing is effective for approximating the global minimum of the negative log-likelihood in a large search space (Belisle, 1992). Since the algorithm decreases the search area with increasing numbers of iterations, we re-ran the procedure with an additional 100 000 iterations using the estimates from the first run as starting values in order to avoid being trapped in a local minima. Models were compared with the small sample unbiased Akaike Information Criterion (AICc; Burnham and Anderson 2002), and the relationships between predicted and observed values were summarized by calculating the percent of deviance explained by each model (analogous with the proportion of variance in classical regression). We also compared our models to two 'null' models that assume either a constant seedling density per unit area ($R_i = \mu$) or that seedling densities depend solely on substrate favourability and not on parent tree distributions ($R_i = \mu F_i$). We estimated standard errors of model parameters, i.e. standard deviations of the sampling distributions, from the inverse Hessian matrix, evaluated at the maximum likelihood parameter estimates (Ridout, 2009).

Table 1. Estimated parameters for six models of seedling dispersal. Bolded values denote most strongly supported model, i.e. lowest AICc and highest % deviance explained. SEs are denoted in parentheses and are not reported for normalization constant, which was not directly estimated in the likelihood function (see text for details). For each model we also calculated the absolute difference between the observed and predicted number of seedlings in our study plots.

Model	STR/μ	X_0/φ	X_b/γ	η	A	θ	β	|obs. − pred. seedlings |	AICc	% deviance explained
**Isotropic lognormal** ($\boldsymbol{\beta = 0}$)	**1 045.8 (272.6)**	**0.67 (0.08)**	**1.81 (0.02)**	**6 011.6**				**59**	**867.5**	**70.2**
Isotropic lognormal	1 041.4 (164.4)	1.46 (0.15)	1.66 (0.02)	10 121.4			0.07 (0.03)	28	885.5	69.5
Anisotropic lognormal	0.07 (<0.01)	20.02 (1.60)	1.48 (0.06)	6.06	2.41 (0.06)	0.29 (<0.01)	0.68 (0.02)	27	961.3	67.0
Substrate only	0.16 (<0.01)							21	1 128.4	61.1
Anisotropic Weibull	<0.01 (<0.01)	0.98 (0.01)	0.28 (<0.01)	0.01	0.08 (<0.01)	1.19 (0.04)	0.12 (<0.01)	98	1 167.7	59.7
Isotropic Weibull	425.4 (255.2)	0.98 (0.03)	0.37 (0.02)	782.1			0.49 (0.21)	325	1 269.4	55.9
Null model	0.05 (0.01)							0	2 827.9	

2.3 Results of seedling dispersal models

Lognormal models were more strongly supported than Weibull models and explained the most deviance in observed seedling densities (Table 1). The spatial distribution of parents was also important (substrate only model versus lognormal models, Table 1), but the orientation of parents relative to dispersal kernels (i.e. anisotropy) did not improve model support (AICc of the isotropic compared to the anisotropic models, Table 1).

The exponent β, which scales seed production with parent tree height, was 0.07 for the isotopic lognormal model, which is considerably less than the expected value of 3 derived from diameter-height relationships (Enquist *et al.*, 1999). Thus, we compared our most strongly supported model (isotropic lognormal) to one where seedling production did not vary with parent tree height, i.e. β was fixed at zero and $1 \leq \text{STR} < \infty$. A model without the effect of parent tree height was by far most strongly supported among our set of candidate models (Table 1). Seedling densities declined exponentially with increasing distance from parent trees, with the highest densities occurring less than 10 m from parent trees (Fig. 1). The most favourable substrate for seedling establishment was *Agrostis-Festuca*

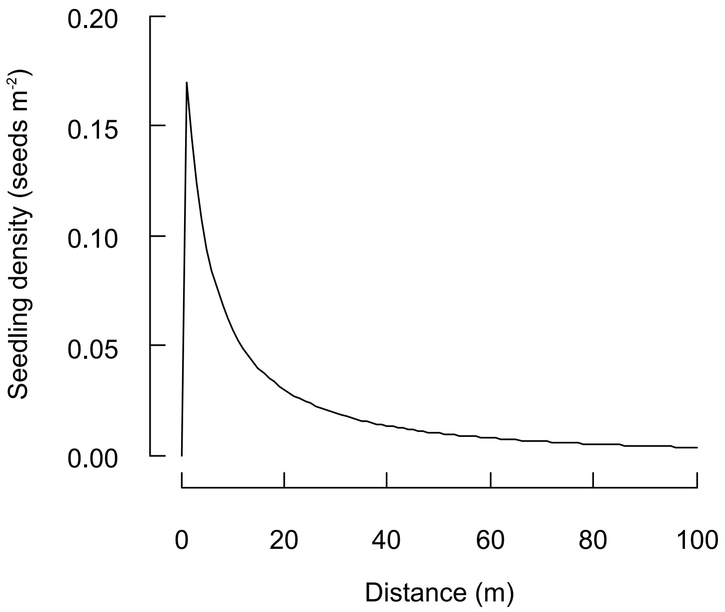

Figure 1. Seedling density (seeds m^{-2}) dispersed by an adult *Betula pubescens* predicted with the most strongly supported dispersal model (isotropic lognormal with seedling density independent of parent tree height).

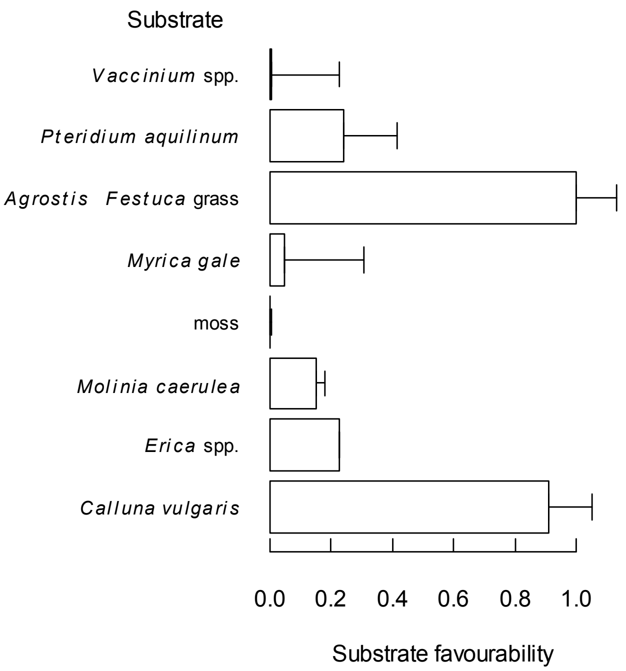

Figure 2. Mean (+SE) for substrate favourability estimated with dispersal model employing isotropic lognormal kernel.

grassland, although *Calluna vulgaris* was similarly favourable (overlapping SEs, Fig. 2). *Vaccinium* spp. (predominantly *V. myrtillus*) were among the least favourable substrates and this may be due to the fact that these species are associated with low levels of understorey light penetration during moorland succession (Hester *et al.*, 1991), which subsequently inhibits the establishment of *B. pubescens* seedlings (Atkinson, 1992). Extremely low favorability of moss contrasts with the findings of Kinnaird (1974), but the relatively high susceptibility of exposed individuals to browsing on these substrates may explain their absence.

3 Juvenile growth and survival

3.1 Derivation of size-based matrix model

We estimated growth and survival of juvenile birch using five measurements conducted at 2-year intervals of the numbers of juvenile trees in three size classes in 60 permanent plots at Creag Meagaidh. Our approach involved parametrizing a matrix population model (**A**) from the abundance of juvenile trees in each height

tier i at time t ($\mathbf{n_t}$) to determine growth and survival of juvenile birch:

$$\mathbf{A} = \begin{bmatrix} s_1 - g_1 & 0 & 0 \\ g_1 & s_2 - g_2 & 0 \\ 0 & g_2 & s_3 \end{bmatrix}, \tag{8}$$

$$\mathbf{n_{t+1}} = \mathbf{A}\mathbf{n_t}, \tag{9}$$

where $\mathbf{n_t}^{\mathrm{T}} = [\ N_1 \quad N_2 \quad N_3\ ]$, N_i is the number of live trees in each plot in height tier i, with tiers 1, 2, and 3 respectively corresponding with heights of 0 – 2, 2 – 3, and >3 m, s_i is the proportion of trees in height tier i that survive from interval t to $t+1$ and g_i is the proportion of surviving individuals that enter the taller height tier, such that $g_i \leq s_i$. We sampled s_3 from a normal distribution with $\mu = 0.975$ and $\sigma^2 = 0.003$ (see Section 4).

One limitation with our approach is that Eq. 9 does not allow new trees to establish within plots. We did not allow s_3 to contribute to N_1 directly since our plots were only 100 × 2 m, and therefore, a large proportion of established individuals are derived from parent trees outside of plots (i.e. given the dispersal distances of adult birch, see Section 2). To overcome this challenge, we mapped all adult trees (>3 m) within 300 m of each plot (n = 12 581 parent trees) in November 2009. We then applied our most strongly supported model of juvenile dispersal to predict the total number of juvenile trees <2 m in height that would be expected to occur within each plot (N_T), given the dominant vegetation ground cover recorded within each plot in 2009.

The total number of juvenile trees predicted by the dispersal kernel N_T is a function of the number of new trees recruited at each time period (i.e. N_A) and their survival (s_1) and growth (g_1) from the 0 – 2 m height tier from time t to $t+1$. N_T will include juvenile trees of age 1 to T, where T is the maximum age that a juvenile can reach in the 0 – 2 m height tier. We assessed T from 154 juvenile trees <2 m tall that we felled in 2008 across Creag Meagaidh, and from which we cut thin (ca. 5 mm) basal sections that we sanded. Based upon counts of annual growth rings using an optical microscope (Leica Microsystems GmbH, Wetzlar, Germany), T was equal to 27 years. At a given time period t, the number of trees that are newly arrived will therefore be equal to N_A, and $N_A(s_1' - g_1')^1$ for trees that arrived in the previous year $t-1$, where $s_1^* = \sqrt{s_1}$ and $g_1^* = 1 - \sqrt{g_1}$ in order to convert survival and growth estimates to annual rates. In other words, we

calculated the number of trees that arrived in the previous year × their probability of survival to the second year – the number that grew into the taller height tier, and extending this series to the full 27 years:

$$\begin{aligned} N_T &= N_A(s_1^* - g_1^*)^0 + N_A(s_1^* - g_1^*)^1 + N_A(s_1^* - g_1^*)^2 + \ldots N_A(s_1^* - g_1^*)^{27} \\ &= N_A\left[(s_1^* - g_1^*)^0 + (s_1^* - g_1^*)^1 + \ldots (s_1^* - g_1^*)^{27}\right] \\ &= N_A \sum_{n=0}^{27} (s_1^* - g_1^*)^n. \end{aligned} \tag{10}$$

Eq. 10 is equivalent to the expanded form of the finite geometric Maclaurin series and can be simplified as:

$$\begin{aligned} N_T &= N_A\left[\frac{1-(s_1^* - g_1^*)^{28}}{1-(s_1^* - g_1^*)}\right] \\ N_A &= N_T\left[\frac{1-(s_1^* - g_1^*)}{1-(s_1^* - g_1^*)^{28}}\right]. \end{aligned} \tag{11}$$

Since a single N_T was estimated for each plot based on surrounding adult trees, implicit in Eq. 11 is the assumption that N_A is constant among years. We then added $\mathbf{b}^{\mathrm{T}} = [\ N_A \quad 0 \quad 0\ ]$ to $\mathbf{An_t}$ (Eq. 9):

$$\mathbf{n_{t+1}} = \mathbf{An_t} + \mathbf{b}, \tag{12}$$

where N_A is defined by Eq. 11, for which N_T is a known constant and s_1 is estimated.

3.2 Incorporation of deer browsing into matrix models

The proportion of browsed trees ($D_{1,j,t}$) in a given plot j at time t in the 0 – 2 m height tier is a logical predictor of the impacts of deer, but unfortunately, depends on the number of trees present in j. For example, the absence of browsed trees ($B_{1,j,t}$) in plot j at time t may not necessarily arise because the probability of trees being browsed ($p_{1,j,t}$) in j is zero. Rather, $B_{1,j,t}$ can be equal to zero because there are no trees in that plot ($N_{1,j,t}$), and so there is no way of knowing whether there would be browsed trees given the opportunity. A similar situation may arise when all trees are browsed in a plot, but $N_{1,j,t}$ is very small, i.e. = 1. The high value of $D_{1,j,t}$ (= 1) may not reflect the true probability of browsing

activity ($p_{1,j,t}$) since there is only one 'trial of whether browsing occurred in that plot. To account for this uncertainty, we modelled $p_{1,j,t}$ from a binomial process based on the total number of trees in a plot in the 0 – 2 m height range ($N_{1,j,t}$), which incorporates the entire vertical range of deer browsing, and the number that were browsed ($B_{1,j,t}$):

$$\begin{aligned} B_{1,j,t} &\sim B(p_{1,j,t}, N_{1,j,t}), \\ \text{logit}(p_{1,j,t}) &= \psi + \upsilon_j + \upsilon_t, \end{aligned} \tag{13}$$

where $p_{1,j,t}$ was modelled based on the mean probability of a tree being browsed across the landscape (ψ), and υ_j and υ_t respectively accounted for spatial and temporal variation, which were each $\sim N(0, \tau_\upsilon)$ with a separately estimated τ_υ. Eq. 13 is particularly ideal for our simulation model since it allows us to use a mean landscape-level probability of browsing with random variation across the landscape and among years, which can capture plot-level variation in factors such as microtopography and climate-related foraging activity.

We used $p_{1,j,t}$ to incorporate the effects of deer browsing into our matrix model (Eq. 12), specifically in terms of the survival (s_1) and growth (g_1) of juvenile trees in the 0 – 2 m height tier. We defined the effects of deer through the coefficient $d'_{1,j,t}$, and fitted linear and non-linear relationships whereby:

$$d'_{1,j,t} = 1 - dp_{1,j,t}, \tag{14}$$

$$d'_{1,j,t} = e^{dp_{1,j,t}}, \tag{15}$$

$d'_{1,j,t}$ is the estimated 'strength' of the deer effect in plot j at time t and ranges between 0 and 1, and d is the effect of deer browsing, and $p_{1,j,t}$ is the probability of trees being browsed in each plot j at time t in the 0 – 2 m height tier. We multiplied the survival and forward transition probabilities of juvenile trees in the browse layer (i.e. s_1 and g_1) by d'. To constrain d' to values between 0 and 1, we assumed that $d \in [0, 1]$ for the linear model (Eq. 14), and that $d \in (-\infty, 0]$ for the exponential model (Eq. 15). For all other height tiers outside of the browse layer, we assumed no effect of deer, such that:

$$d'_{i,j,t} = 1 \text{ for } i \in \{2, 3\}.$$

Estimation of annual seed rain N_A from N_T in each plot j at time t should also be affected by browsing. Converting the effect of deer to an annual rate $d^*_{1,j}$, where $d^*_{1,j} = \sqrt{(d'_{1,j})}$, leads to:

$$\begin{aligned} N_{T,j,t} &= N_{A,j}[d^*_{1,j}(s^*_1 - g^*_1)]^0 - N_{A,j}[d^*_{1,j}(s^*_1 - g^*_1)]^1 + N_{A,j}[d^*_{1,j}(s^*_1 - g^*_1)]^2 \\ &+ \ldots N_{A,j}[d^*_{1,j}(s^*_1 - g^*_1)]^{27}, \end{aligned} \tag{16}$$

such that the survival s^*_1 and growth g^*_1 of juvenile trees depends on the effect of deer browsing $d^*_{1,j}$ in plot j. We assumed that the mean effect of temporal variation in deer browsing υ_t within a given plot j was equal to zero (Eq. 13), allowing us to simplify $d'_{1,j,t}$ to $d'_{1,j}$ in Eqs 14 and 15. This assumption was important because it allowed us to then re-apply the Maclaurin series simplification. Given that our observed data also correspond to changes in juvenile tree counts over two-year intervals, we calculated $N_{A,j,t}$ over two years and ensured that 1-year old seedlings are affected by deer:

$$N_{A,j} = N_{T,j}\frac{1 - d^*_{1,j}(s^*_1 - g^*_1)}{1 - [d^*_{1,j}(s^*_1 - g^*_1)]^{28}}\left[1 + d^*_{1,j}(s^*_1 - g^*_1)\right],$$

which accounts for the number of newly arrived seedlings at time t and the number that survived from the previous year, which are affected by deer browsing $(d'_{1,j})$. Our final model then took the following form:

$$\begin{bmatrix} N_{1,j,t+1} \\ N_{2,j,t+1} \\ N_{3,j,t+1} \end{bmatrix} = \begin{bmatrix} d'_{1,j,t}(s_1 - g_1) & 0 & 0 \\ d'_{1,j,t}g_1 & s_2 - g_2 & 0 \\ 0 & g_2 & s_3 \end{bmatrix} \begin{bmatrix} N_{1,j,t} \\ N_{2,j,t} \\ N_{3,j,t} \end{bmatrix} + \begin{bmatrix} N_{A,j} \\ 0 \\ 0 \end{bmatrix}. \tag{17}$$

3.3 Dataset preparation

Given the potential implications of predictions derived from our model for land management across the U.K., we attempt to be fully transparent in how we prepared the Creag Meagaidh National Nature Reserve (CM NNR) dataset prior to model estimation. Staff changes during the measurement of monitoring transects are likely to have introduced inaccuracies into the dataset (R. Richardson pers. comm.). In the Supporting Information, we provide both the original dataset, as well as the modified version described here, as `*.txt` files. Briefly, we prepared the dataset prior to model estimation as follows:

1. We obtained raw counts of trees along six transects in originally four rather than three height tiers, corresponding with 0 – 1, 1 – 2, 2 – 3, and >3 m height tiers. We merged the 0 – 1 and 1 – 2 height tiers to collectively model trees in the 'browse' layer. This approach minimized our concerns about measurement errors we observed in the dataset, whereby trees appeared to 'skip' height tiers. For example, 0 and 1 tree(s) in the first and second height tiers of plot 3 on transect 2 in 2002 became 1 and 3 trees in these height tiers in 2004. This indicated that at least two trees grew >0.5 m yr^{-1} in height immediately upon establishing within the plot. However, this growth rate is highly unlikely based on measurements of juvenile height growth adjacent to two of the transects in 2008 (n = 127), i.e. mean distance (± SE) from the first terminal bud scar immediately beneath the apical meristem to the apical meristem was 0.05 ± 0.01 cm yr^{-1} (A. J. Tanentzap unpub. data).

2. We removed 31 plot measurements from our dataset because of concerns over consistency in recording methods. Measurements were along transects 2, 4, 7, and 8, and all were either from 2002 or 2004. Our concern was that the number of trees damaged by deer in each of these plots was inflated because any sign of browsing was used to classify a tree as damaged, rather than only classifying trees as damaged if their leader stems were broken or stripped of bark. Specifically, the mean proportion of damaged trees in these plots (± SE) was 72 ± 3% compared with 5 ± 1% in the same plots averaged over the other measurement years. There were no changes in deer culling or mortality over this period that would have precipitated the dramatic reduction in browse damage. Rather, CM NNR has pointed to staff changes during this period as the cause for this discrepancy (R. Richardson pers. comm.).

3. We removed measurements post-2005 for two plots. Plot 1 along transect 2 was not recorded after 2006 since juvenile tree densities were judged by CM NNR staff as reaching a desirable level. Similarly, plot 8 on transect 3 was recorded in 2010 but not in 2008. In 2006, both plots had unusually high numbers of trees >3 m (42 and 17, respectively), representing increases of 35 and 17, respectively, over the previous two years. We also removed these outliers from the dataset.

4. We made 38 other small corrections to the dataset. These changes were primarily intended to correct data entry or inconsistencies between time periods ($n = 16$), prevent trees from jumping from the $0-2$ m to >3 m height tier within two years by re-assigning trees to the $2-3$ m height tier ($n = 17$ instances), and reduce the number of damaged trees in plots with ≤ 4 trees in 2002 to zero as we were fairly confident of over-estimation in these cases ($n = 5$).

3.4 Derivation of model likelihood function

We were interested in inversely estimating the elements of matrix $\mathbf{A}$ (Eq. 8) given the vectors $\mathbf{n_t}$ and $\mathbf{b}$. Eq. 8 is deterministic because $\mathbf{n_1}$ is sufficient to describe the population dynamics of our entire time series. By introducing stochastic components into Eq. 17 to account for measurement (or 'observation') error, we were able to derive a probability model that could be used for deriving a likelihood function from which to estimate $\mathbf{A}$. We also allowed for error ($\epsilon_{i,j,t}$) in the tree counts $N_{i,j,t+1}$ within each height tier i at time t in plot j and assumed that:

$$\begin{aligned} N_{i,j,t+1} &\sim Poisson(\lambda_{i,j,t}) \\ \log(\lambda_{i,j,t}) &= \log(\mathbf{A_i n_{i,j,t}} + \mathbf{b_{i,j}}) + \epsilon_{i,j,t}, \end{aligned} \tag{18}$$

where i is a given height tier and thus the ith row of matrix $\mathbf{A_i}$ and ith element of vectors $\mathbf{n_{i,j,t}}$ and $\mathbf{b_{i,j}}$. We also assumed that $\epsilon_{i,j,t}$ was $\sim N(0, \tau_\epsilon)$ and $\upsilon_{1,j,t}$ was $\sim \log N(0, \tau_\upsilon)$. The full conditional log-likelihood of observing $N_{i,j,t+1}$ trees in height tier i at time $t+1$ in plot j could then be written as the Poisson probability mass function:

$$\begin{aligned} \log L(N_{i,j,t+1}|d'_{i,j,t}, s_i, g_i) &= \sum_{j=1}^{n} \sum_{t=t+1}^{t+y} \sum_{i=1}^{3} [e^{\epsilon_{i,j,t}}(\mathbf{A_i n_{i,j,t}} + \mathbf{b_{i,j}}) \\ &+ N_{i,j,t+1} e^{\epsilon_{i,j,t}} \log(\mathbf{A_i n_{i,j,t}} + \mathbf{b_{i,j}})]. \end{aligned} \tag{19}$$

3.5 Parameter estimation

We used a hierarchical Bayesian framework to estimate Eq. 19 (Gelman and Hill, 2007). Our data correspond to a hierarchical design because trees were sampled within three height tiers within each plot over multiple years. Bayesian methods

are particularly attractive as they allow for robust estimation of both parameters and their confidence intervals. These methods thus have the advantage of propagating uncertainty and both temporal and spatial variation into predictions from our simulation model.

We introduced variation into estimates of survival and recruitment through the specification of prior distributions for model parameters. We allowed both survival and growth to vary between height tiers i and among measurement periods t:

$$\text{logit}(s_{i,t}) = \alpha_i + \upsilon_{s_t},$$

$$\text{logit}(g_{i,t}) = \beta_i + \upsilon_{g_t},$$

where $s_{i,t}$ is the mean probability of survival in height tier i from time t to $t+1$, $g_{i,t}$ is the mean probability of transitioning from height tier i to a taller tier at time t, α and β are estimated parameters, and υ_{s_t} and υ_{g_t} are the variance among years for s_i and g_i, respectively, and each is $\sim N(0, \tau_t)$, with a separately estimated τ_t. To ensure $s_{i,t}$ and $g_{i,t} \in [0, 1]$, we used the logit function, which assumed that survival and growth increased exponentially. Mean survival and growth probabilities could simply be described by α and β, respectively, since the mean effect of temporal variation was zero. We then used these mean values for the $0-2$ m height tier (i.e. time invariant s_1 and g_1) to calculate seed rain $N_{A,j,t}$ (Eq. 16) for the two-year period between plot measurements, converting estimates to annual rates using known effects for survival, growth, and deer browsing in that period. $N_{A,j}$ at a given time t thus took the following form during parameter estimation, which incorporated some degree of temporal variation by allowing for newly arrived, 1-year old seedlings to be affected by the annual survival ($s^*_{1,t} = \sqrt{s_{1,t}}$), growth ($g^*_{1,t} = 1 - \sqrt{1 - g_{1,t}}$), and effect of deer at that specific time period t ($d^*_{j,t}$):

$$N_{A,j,t} \quad = \quad N_{T,j} \frac{1 - d^*_{1,t}(s^*_1 - g^*_1)}{1 - [d^*_{1,t}(s^*_1 - g^*_1)]^{28}} \left[1 + d^*_{j,t}(s^*_{1,t} - g^*_{1,t})\right].$$

We specified relatively uninformative priors for model parameters. We assumed that all estimated means (ψ, α, β, d) were drawn from normal distributions with $\mu = 0$ and $\sigma^2 = 100$, though we used a truncated normal distribution for d depending on the model form (see Section 3.2). Variance parameters $(\tau_\epsilon, \tau_\upsilon, \tau_t)$ were $\sim U(0, 100)$.

We fit our model to the corrected dataset (see Section 3.3) using Markov chain Monte Carlo (MCMC) sampling by calling `JAGS` $v3.1.0$ (Plummer, 2011) from `R` $v2.13$ (R Development Core Team, 2011) with the `R2jags` package (Su and Yajima, 2011). Five MCMC chains of 1 000 000 iterations were simulated for each model, with a burn-in period of 1 000 000 runs. We sampled each chain 160 times to generate a posterior density for each parameter comprised of 800 simulations, and calculated posterior means and 95% credible intervals (CIs) for each parameter. Convergence was assessed visually by chain traces and by calculating the potential scale reduction factor, $\hat{R}$, for each parameter from the 800 simulation subsets. $\hat{R}$ predicts the extent to which a parameter's confidence intervals will be reduced if models are run forever; all our $\hat{R}$ values were $\leq$1.1, which is considered acceptable (Gelman and Hill, 2007). We also ensured that the effective number of simulation draws, n_{eff}, a measure of the independence amongst the subset of 800 simulations, always exceeded 100 (Gelman and Hill, 2007).

The `JAGS` code for the model is included in Appendix S1. We also tried fitting our model to the original dataset, but the model failed to converge given the discrepancies where trees appeared to 'jump' height tiers, e.g. trees from the 0 – 2 m tier were >3 m tall after two years (see Section 3.3).

3.6 Model assessment

We used 95% CIs to test the significance of model effects. Specifically, we compared 95% CIs for survival and growth (i.e. forward transition) probabilities between height tiers to test whether $s_{1,t} = s_{2,t}$ and $g_{1,t} = g_{2,t}$, i.e. non-overlapping intervals. We also used this approach to determine whether deer affected juvenile trees. If 95% CIs for the estimated effect of browsing d overlapped zero (= overlapping with 1 for d'), we concluded that deer did not affect juvenile tree transitions. Finally, we compared models fitted with linear versus non-linear effects of deer browsing (Eqs 14 *vs* 15) by comparing 95% CIs for the estimated model deviance. Since both models have similar numbers of parameters, we chose the model with the lower deviance as more strongly supported and for use in our simulation modelling.

We compared predicted and observed tree counts in each height tier in each plot at each time period, and the proportion of trees that were browsed in the 0 – 2 m height. To assess model fit, we calculated a Bayesian R^2 at the level of our observed data, which is synonymous to the proportion of variance in classical

linear regression (Gelman and Pardoe, 2006):

$$R^2 = 1 - \frac{E(V_{k=1}^{K} \epsilon_k)}{E(V_{k=1}^{K} y_k)},$$

where E is the posterior mean, V is the variance, ϵ_k are the residual errors of the K observations, and y_k are the predicted values for the response variable.

3.7 Conversion of parameter estimates to annual rates

Our dataset for juvenile trees corresponded to two-year intervals. Therefore, we took the square root of estimated survival values and the effect of deer to convert estimates to annual rates. For growth, we calculated g as $1 - \sqrt{1-g}$.

3.8 Results of juvenile growth and survival models

The forward transition probability (i.e. growth) of juvenile trees, and their susceptibility to deer browsing, controlled population size structure. Juvenile survival was high (mean within 0 – 2 and 2 – 3 m height tiers across years = 0.98 yr^{-1}; 95% CIs: 0.70 – 1.00 yr^{-1}), but few trees transitioned among height classes (mean across tiers and years = 0.04 yr^{-1}; 95% CIs: <0.01 – 0.17 yr^{-1}; Table 2). Growth did however vary among years and height tiers, while survival was invariant (95% CIs, Table 2).

Deer browsing reduced annual growth and survival by an average of 10% (mean effect of $d' = 0.90$ yr^{-1}; 95% CIs = 0.52 – 1.00, depending on proportion of trees browsed; Fig. 3). Although there was no difference between linear and non-linear models of browsing (overlapping 95% CIs for deviance; M1 *vs* M2, Table 2), we adopted the non-linear model because empirical support for it is widespread in the literature (e.g. Tremblay *et al.* 2006; Gill and Morgan 2010; Koh *et al.* 2010; Koda and Fujita 2011). Consequently, few juveniles survived or transitioned to taller height tiers when >50% of individuals were browsed (Fig. 3). The model explained most of the variation in the observed data (Bayesian $R^2 \geq 0.90$; Fig 4), though there was considerably more unexplained variation in the probability of browsed trees when we only considered observed values of ≤ 0.30 (Bayesian $R^2 = 0.49$). One explanation is that browsing decisions are also influenced by factors such as the presence of other browse species, topography, sex and age

structure of deer populations, and season (Palmer *et al.*, 2003; Bee *et al.*, 2008, 2010), in addition to the density of birch trees at a given site.

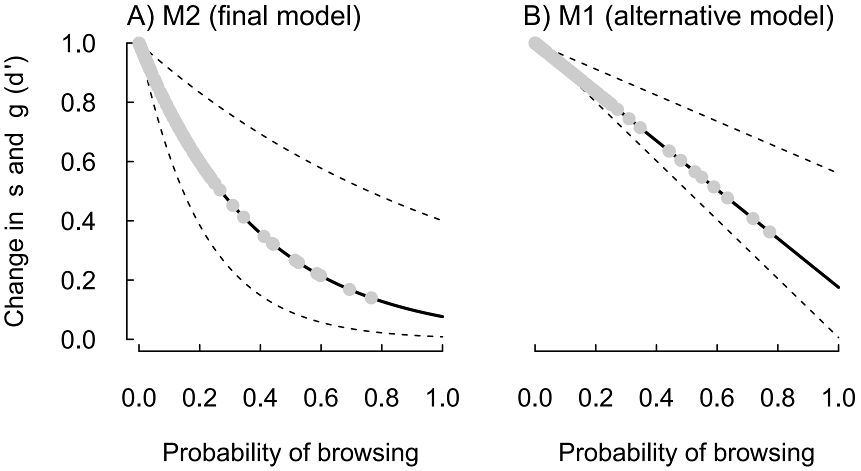

Figure 3. Predicted relationship between probability of a tree being browsed by deer and coefficient of deer effect d', which reduces survival (s) and growth (g) of juvenile trees. Points represent means of model predicted values. Dashed lines are 95% CIs.

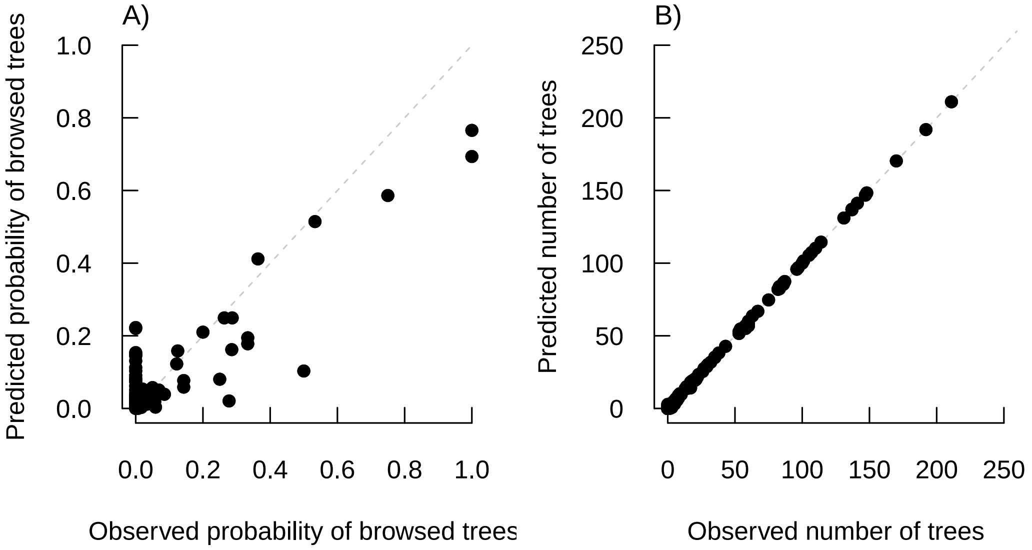

Figure 4. Comparisons of model predictions versus observed values for juvenile matrix model with non-linear effects of deer. A) Probability of trees being browsed in a plot (Bayesian $R^2 = 0.90$). B) Juvenile tree counts (Probability of trees being browsed in a plot (Bayesian $R^2 = 0.99$). Solid line represents 1:1 relationship.

Table 2: Estimated parameters (95% CIs) for two models of probability of juvenile survival and growth (i.e. forward transition), with values representing changes over two-year period. M1: Model estimated with linear effects of deer browsing; M2: Final model used in simulation model and with non-linear effects of deer. Models were compared based on deviance, with both models equally supported (i.e. overlapping 95% CIs for deviance). A model fit with the uncorrected dataset failed to converge.

Estimated parameter	M1	M2
Mean survival in 0 – 2 m height tier (α_1)	76.1 (4.34 – 211.0)	82.9 (5.55 – 237.0)
Mean survival in 2 – 3 m height tier (α_2)	84.5 (2.03–243.0)	82.9 (2.13 – 225.0)
Mean growth in 0 – 2 m height tier (β_1)	-5.04 (-7.10 – -3.64)	-5.04 (-7.23 – -3.52)
Mean growth in 2 – 3 m height tier (β_2)	-2.22 (-4.48 – -0.60)	-2.27 (-4.55 – -0.46)
Effect of deer browsing (d)	0.83 (0.44 – 0.99)	-2.57 (-4.77 – -0.92)
Mean probability of deer browsing (ψ)	-2.35 (-4.74 – -0.10)	-3.04 (-4.94 – -1.05)
Inter-plot variance in probability of browsing (τ_{v_j})	2.34 (1.65 – 3.36)	2.29 (1.62 – 3.18)
Temporal variance in probability of browsing (τ_{v_t})	4.14 (1.28 – 13.2)	3.20 (1.09 – 10.1)
Temporal variance in survival ($\tau_{s,t}$)	39.2 (1.80 – 96.7)	40.0 (1.74 – 95.1)
Temporal variance in growth ($\tau_{g,t}$)	1.47 (0.23 – 6.18)	1.50 (0.25 – 5.31)
Residual variance (τ_ϵ)	1.03 (0.86 – 1.22)	1.04 (0.87 – 1.23)
Survival in 0 – 2 m height tier, 2002 – 2004 ($s_{1,1}$)	1.00 (0.97 – 1.00)	1.00 (1.00 – 1.00)
Survival in 0 – 2 m height tier, 2004 – 2006 ($s_{1,2}$)	1.00 (0.99 – 1.00)	1.00 (0.99 – 1.00)
Survival in 0 – 2 m height tier, 2006 – 2008 ($s_{1,3}$)	0.85 (0.45 – 1.00)	0.91 (0.46 – 1.00)
Survival in 0 – 2 m height tier, 2008 – 2010 ($s_{1,4}$)	0.99 (0.76 – 1.00)	0.99 (0.83 – 1.00)

Survival in 2 – 3 m height tier, 2002 – 2004 ($s_{2,1}$)	1.00 (1.00 – 1.00)	1.00 (0.99 – 1.00)
Survival in 2 – 3 m height tier, 2004 – 2006 ($s_{2,2}$)	1.00 (0.99 – 1.00)	1.00 (1.00 – 1.00)
Survival in 2 – 3 m height tier, 2006 – 2008 ($s_{2,3}$)	0.86 (0.20 – 1.00)	0.88 (0.21 – 1.00)
Survival in 2 – 3 m height tier, 2008 – 2010 ($s_{2,4}$)	0.99 (0.98 – 1.00)	0.99 (0.98 – 1.00)
Growth in 0 – 2 m height tier, 2002 – 2004 ($g_{1,1}$)	0.01 (<0.01 – 0.03)	0.01 (<0.01 – 0.03)
Growth in 0 – 2 m height tier, 2004 – 2006 ($g_{1,2}$)	0.01 (<0.01 – 0.02)	0.01 (<0.01 – 0.02)
Growth in 0 – 2 m height tier, 2006 – 2008 ($g_{1,3}$)	<0.01 (<0.01 – 0.01)	<0.01 (<0.01 – 0.01)
Growth in 0 – 2 m height tier, 2008 – 2010 ($g_{1,4}$)	0.01 (0.01 – 0.02)	0.01 (0.01 – 0.03)
Growth in 2 – 3 m height tier, 2002 – 2004 ($g_{2,1}$)	0.13 (0.02 – 0.38)	0.13 (0.02 – 0.37)
Growth in 2 – 3 m height tier, 2004 – 2006 ($g_{2,2}$)	0.15 (0.04 – 0.36)	0.16 (0.05 – 0.36)
Growth in 2 – 3 m height tier, 2006 – 2008 ($g_{2,3}$)	0.06 (0.01 – 0.16)	0.06 (<0.01 – 0.15)
Growth in 2 – 3 m height tier, 2008 – 2010 ($g_{2,4}$)	0.19 (0.07 – 0.39)	0.19 (0.07 – 0.38)
Deviance	1 224.0 (1 176.1 – 1 275.6)	1 224.6 (1 177.6 – 1 275.4)

4 Adult growth and mortality

4.1 Adult growth

We used measurements of adult trees at the Corrour Estate to estimate annual radial growth (D_G, mm yr^{-1}) of adult birch trees from their diameter at breast

height (D, cm; Eq. 20; Canham *et al.* 2004):

$$D_G = D_M e^{-\frac{[log(D) - log(X_0)]^2}{2X_b^2}}, \tag{20}$$

where D_M is the maximum potential radial growth rate (mm yr^{-1}), X_0 is the diameter at which D_M occurs (cm), and X_b controls the steepness of the function. We assumed a Gaussian error structure. We estimated rates of mean annual height growth (H_G, m yr^{-1}) from measurements of D_G using the relationship between D and the height of each tree (H, m). We modelled the relationship between height and diameter both as a power function (Eq. 21) and as an asymptotic function (Eq. 22; Russo *et al.* 2007):

$$H = \gamma D^{\delta}, \tag{21}$$

$$H = H_M - e^{aD^b}, \tag{22}$$

where γ, δ, a, and b are estimated parameters and H_M is the estimated maximum potential tree height. We assumed a lognormal error structure for these models, and estimated H_G as the first-order differential of either Eqs 21 or 22.

We estimated model parameters using maximum-likelihood methods. The negative log-likelihood of each model was minimized using 100 000 iterations of the simulated annealing algorithm for the `optim` function (R Development Core Team, 2011). Simulated annealing is effective for approximating the global minimum of the negative log-likelihood in a large search space (Bélisle, 1992). However, since the algorithm decreases the search area with increasing numbers of iterations, we re-ran the procedure with an additional 100 000 iterations using the estimates from the first run as starting values in order to reduce the possibility of being trapped in a local minimum. We estimated standard errors of model parameters from the inverse Hessian matrix, evaluated at the maximum likelihood parameter estimates (Ridout, 2009). We compared Eq. 20 to a model of mean growth rate ($D_G = \mu$), and Eq. 21 with Eq. 22, using AICc.

4.2 Adult mortality

The most sensible approach for estimating adult mortality (M, %) for our dataset was to utilize the relationship among D_G, M, and the population size distribution, $n(D)$, which occurs at demographic equilibrium (Coomes *et al.*, 2003; Kohyama

et al., 2003; Muller-Landau *et al.*, 2006). For a constant D_G and M:

$$n(D) = \frac{q}{D_G} e^{-\frac{M}{D_G}(D-D_0)}, \tag{23}$$

where q is the annual recruitment rate and D_0 is the mean minimum size of adults (0.68 cm, estimated from Eq. 21 with $H = 3$ m). Eq. 23 represents an exponential size distribution and thus has a probability density function of the form:

$$p(D) = \frac{M}{D_G} e^{-\frac{M}{D_G}(D-D_0)}, \tag{24}$$

where $p(D)$ is the probability distribution for D. We were constrained in the number of other potential approaches for estimating M since we lacked long-term repeated measurements of adult trees or stand structure, and the relatively low densities of mature trees at our site limited the availability of dead adults (*cf.* Wyckoff and Clark 2000).

We used the `fitdistr` function in `R` (R Development Core Team, 2011) to fit an exponential distribution to Eq. 24 with maximum-likelihood estimation. $D - D_0$ was equal to the observed diameters at Corrour and the diameters estimated with either Eq. 21 or 22 for adult trees in Creag Meagaidh seedling plots, where γ and δ in Eq. 21, or a and b in Eq. 22, were randomly drawn from a normal distribution with mean and standard deviation equal to the maximum likelihood estimate fit to the respective equations. The estimated rate of the exponential function was then multiplied by D_G to derive M. We compared the exponential fit to a probability distribution of size structure from kernel density estimation. Kernel density estimation provides a non-parametric prediction of the probability density function of a response variable, and is more appropriate for continuous data than histograms that arbitrarily classify the response variable in data bins. The overall kernel density estimate was the sum of individual Gaussian distributions around each data point (`density` function in `R`, Silverman 1986).

We acknowledge that the assumption that the adult population was in demographic equilibrium was unlikely to be true at our site, and we tested the sensitivity of M to changes in $n(D)$. We simulated growth, recruitment, and mortality, in our adult tree population for 100 and 500 years, after which we re-estimated mortality from Eqs 23 and 24. For each year, we allowed trees to increase in size at a rate of D_G, and randomly removed trees from the population at a rate of M.

We added new trees at the end of each year at rates ranging from $0.5M$ to $2.0M$, where the coefficients of M increased in intervals of 0.1. Sizes of new trees were randomly drawn from a uniform distribution on the range $[D_0, 1.2D_0]$. We re-ran the simulation procedure 1 000 times for each recruitment rate to generate means and standard errors.

4.3 Competition for light and density-dependent mortality in adults

We recognize that as adult trees establish in our model, the canopies of individuals may overlap, resulting in competition for light, and consequently, density-dependent mortality. Since our primary objective was to develop a model of birch invasion that operated over relatively short-time scales in relation to deer management (i.e. <100 years), and not to simulate the dynamics of forest stands, we did not expect high levels of overlap in the canopies of adult trees. Our model is only likely to generate competition for light among adults over long periods (i.e. centuries) and at high levels of seedling survival. However, in order to mitigate the possibility of this issue arising, we predicted the crown diameter of each adult tree from tree height, and removed trees from the simulation when neighbouring individuals overtopped 90% of their crown area (*sensu* Yoda 1963; Westoby 1984).

We predicted crown diameter (C, m) from measurements of tree height (H, m), using the same power and asymptotic functions used to relate H to tree diameter (Eqs 21 and 22). Models were estimated by minimizing the negative log-likelihood of a lognormal distribution using 200 000 iterations of simulated annealing (`optim` function in `R`) and compared using AICc. We estimated standard errors of model parameters from the inverse Hessian matrix, evaluated at the maximum likelihood parameter estimates (Ridout, 2009).

4.4 Results of adult growth and mortality models

Radial growth was invariant with size (AICc of size-based and mean-based models: 44.6 and 42.2, respectively; Fig. 5), consistent with other studies of *Betula* spp. (Ward and Stephens, 1997), including at a nearby site (old-growth stands, Mountford and Peterken 2000). Mean growth rate was 2.66 mm yr^{-1}, compared to a D_G under a size-based model ranging from 2.66 – 2.67 mm yr^{-1} over observed

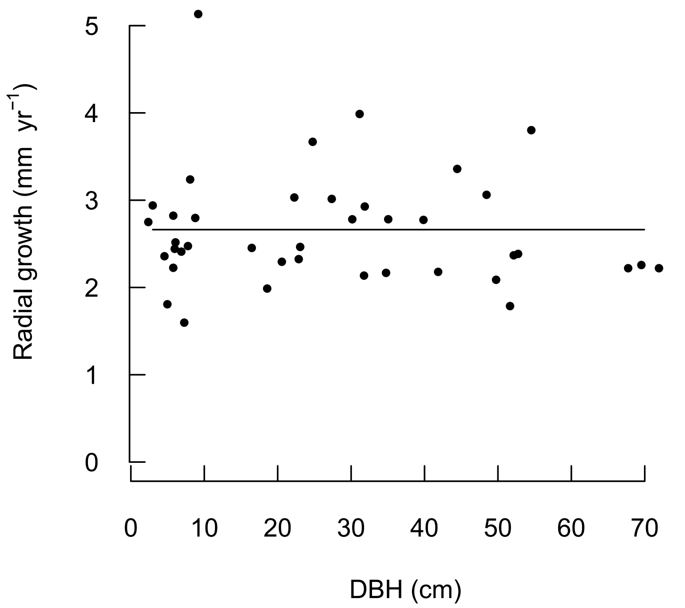

Figure 5. Annual radial growth averaged over 2001 – 2008 for *Betula pubescens* at Corrour Estate. Solid line is the mean model of radial growth (2.66 mm yr^{-1}), which was more strongly supported based on AICc than a size-based model (R^2 = 0.54).

values of D of 2.6 – 72.2 cm. We used the estimate of mean growth rate in all subsequent analyses.

The power function describing the height-diameter relationship was more strongly supported than the asymptotic function and also had fewer parameters (AICc respectively: 207.4 and 210.2). Overall, the power function explained a relatively high proportion of deviance (66%, mean parameter estimates ± S.E.: $\delta = 0.40 \pm 0.05, \gamma = 3.47 \pm 0.48$; Fig. 6A) and the first-order differential was used to predict height growth (i.e. $H_G = \gamma\delta D^{\delta-1} D_G$).

Adult mortality was estimated at a rate of 2.5% yr^{-1} with a standard deviation of 0.3% yr^{-1}. The exponential distribution was a good fit to the empirical probability distribution for trees >40 cm in diameter, estimated from kernel density (Fig. 7). However, we observed fewer trees 20 – 40 cm in diameter than predicted from the exponential distribution (Fig. 8), and this may be due to size-dependent processes operating in these size classes, i.e. herbivory (Coomes *et al.*, 2003). Historically high deer browse pressures at both of our sites have likely led to predominantly bimodal adult size structures (i.e. many small saplings and some

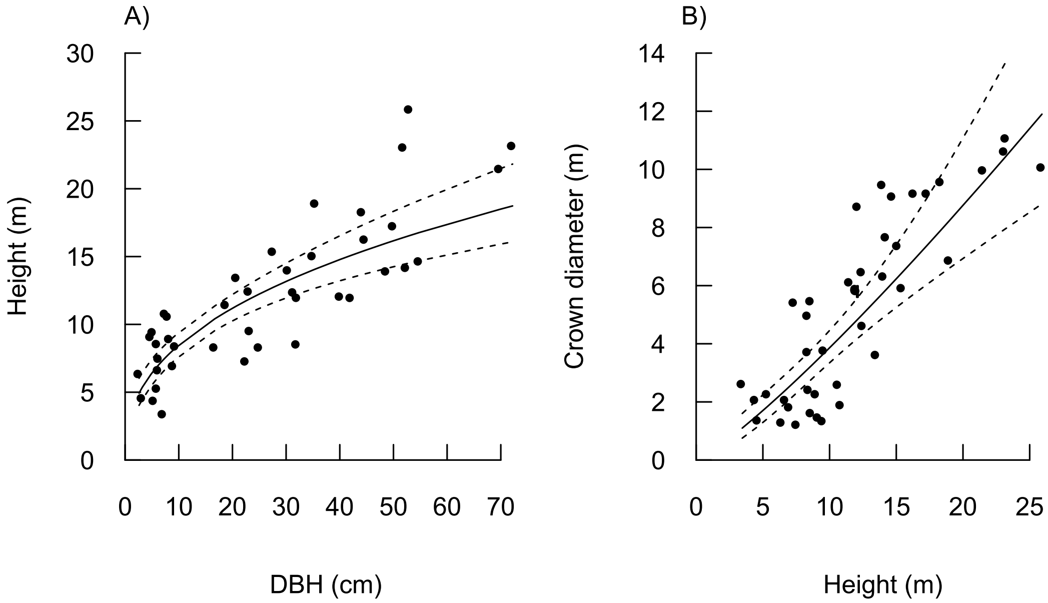

Figure 6. Allometric relationships of adult *Betula pubescens* at Corrour Estate. A) Height-diameter relationship. B) Height-crown diameter relationship. Solid lines represent mean estimates from power functions ± 95 confidence intervals.

large trees) with few saplings able to escape browse pressures into intermediate-sized classes. Although this further suggests that our assumption of demographic equilibrium may be tenuous, changes in size structure arising from simulated demographic disequilibrium have relatively little effect on a constant estimate of mortality, particularly over longer periods (Fig. 8).

The power function to describe height-crown diameter relationships was more strongly supported than the asymptotic model and had one fewer parameter (AICc respectively: 170.4 and 172.7). Mean parameter estimates ± S.E. were: $\delta = 1.18 \pm 0.16, \gamma = 0.28 \pm 0.11$ (percent of deviance explained = 67%; Fig. 6B).

5 Measures of deer browse pressure

5.1 Indicators of deer browse pressure

Staff at Creag Meagaidh National Nature Reserve have continuously recorded the proportion of juvenile *Betula* spp. damaged by deer along with monthly deer population counts in eight sub-catchments since 2002. Damaged juvenile birch

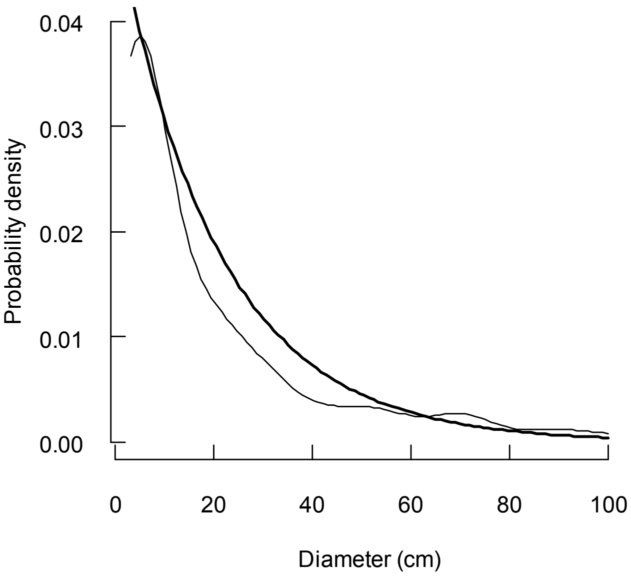

Figure 7. Probability distribution of *Betula pubescens* size structure at Corrour Estate and Creag Meagaidh National Nature Reserve in 2008 predicted with kernel density estimation (thin line) and from an exponential distribution (solid line).

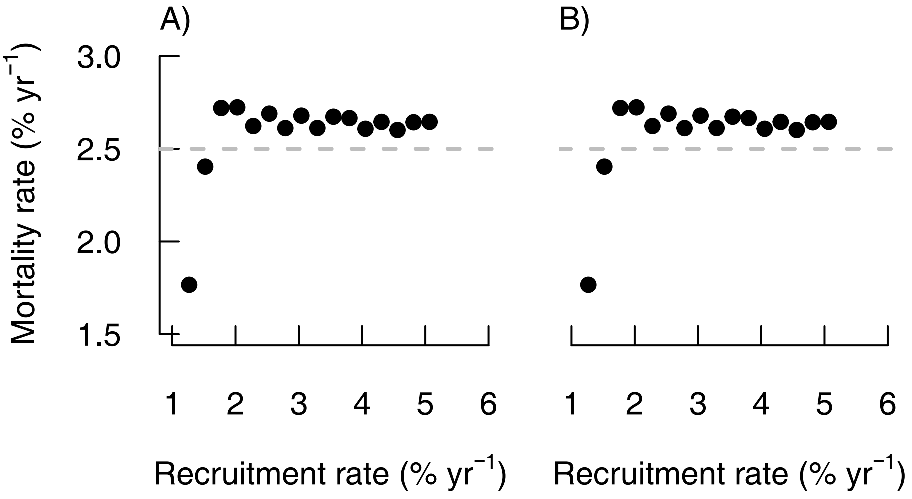

Figure 8. Mean (±SE) estimated mortality rate derived from fitting exponential distribution to simulated *Betula pubescens* size structure at Corrour Estate and Creag Meagaidh National Nature Reserve after A) 100 and B) 500 years at various recruitment rates and with a constant growth rate of 2.66 cm^2. Dashed line denotes estimated mortality rate of 2.5% derived from observed data.

trees were monitored along 16 permanently marked 1-km long transects (hereafter 'tree transects'). Nine transects were located in areas where tree regeneration was expected, while the remaining seven were randomly located throughout the reserve. Each tree transect was measured in May or June every other year from 2002 to 2008, with all the intentionally-positioned transects measured in even years and the randomly-located transects measured in odd years. Methods to estimate the proportion of *Betula* trees damaged by deer along all 16 transects were identical to those used for the six transects that were input in our matrix model. Since the main text describes these methods, we do not repeat them here. Putman *et al.* (2005) detail the methodology for deer population counts and the census zones. Briefly, the total number of deer seen in each of eight counting zones (2.2 – 10.5 km^2) was recorded monthly each year by at least one observer. Observers traversed counting zones by foot over a period of varying length, recording deer numbers from a distance not to disturb herds and risk double counting.

We derived an index of deer density (C, deer km^{-2} observers^{-1} hour^{-1}) for each deer counting zone i by averaging deer counts over a 24-month period (July – June) preceding measurement of the transects. We standardized deer counts (N, deer) for each month j by sampling effort, since the number of observers (O, people) and time spent in each counting zone (T, hours) varied among months and zones:

$$C_{i,j} = \sum_{j=1}^{24} \frac{N_{i,j}}{A_i O_{i,j} T_{i,j}}. \quad (25)$$

Eleven of the sixteen tree transects were entirely located within one counting zone, and we assumed that these transects were associated with the deer density estimated for the respective counting zone. We assigned deer densities to the remaining five tree transects by averaging values for the multiple deer counting zones that a transect traversed.

5.2 Models relating deer damage to animal density

We tested whether deer browse damage could be predicted from the number of deer seen per observer (D) hour using a linear mixed-model with binomial error structure (`lmer` function in `lme4` package in `R`, Bates and Maechler 2009). The number of damaged and undamaged *Betula* trees <3 m tall along each transect were the response variables. We allowed for variation in the model intercept among

different transects and years by including a random effect of both transect and year such that:

$$\text{logit}(p_{ijk}) = a + bD_{ijk} + \upsilon_j + \upsilon_k, \tag{26}$$

where p_{ijk} is the proportion of trees damaged by deer in plot i along transect j in year k, a and b are estimated parameters, and υ_j and υ_k are random effects that account for variance among transects and years, respectively, and are $\sim N(0, \sigma_j)$ and $\sim N(0, \sigma_k)$, where σ_j and σ_k are estimated. We fit the model using maximum likelihood (ML) and tested whether the effect of D was different from zero using a likelihood-ratio test with a χ^2 approximation. We could not use AICc for model inference since it cannot be reliably calculated for mixed effects models estimated with ML (Bolker *et al.*, 2009).

We also tested whether the total density of trees <3 m tall in each plot (N_{ijk}) declined with D by re-fitting the model defined by Eq. 26 with a Poisson error structure and log-link function. N_{ijk} replaced p_{ijk} as the response variable, and we added an individual-level random effect υ_{ijk} to account for the fact that tree counts were overdispersed, i.e. greater variance than predicted by Poisson process (Elston *et al.*, 2001). We compared models fit with and without D using a likelihood-ratio test.

Our model defined by Eq. 26 resembles that used to predict the probability of browsing for juvenile trees, i.e. Eq. 13. However, we could not use estimates of deer density in our matrix model, because this would have necessitated data on deer densities to predict the probability of browsing in the validation of our model, and these data were unavailable prior to 1992 for the eight counting zones (Putman *et al.*, 2005); any predictors used for estimating the probability of browsing in our matrix model must be available in the validation procedure. Additionally, although we accounted for variance among transects and years in both Eq. 13 and Eq. 26, the datasets used to fit these functions are different, so parameter estimates will be similar only if unbiased samples are drawn from the overall population.

5.3 Relationships between deer browsing and density

Increasing deer densities negatively affected juvenile birch trees <3 m tall. Deer browse damage increased with deer density, with strong increases in browse damage above 0.10 deer km^{-2} ($\chi^2 = 27.6$, $p < 0.001$, Fig. 9A). Similarly, the density

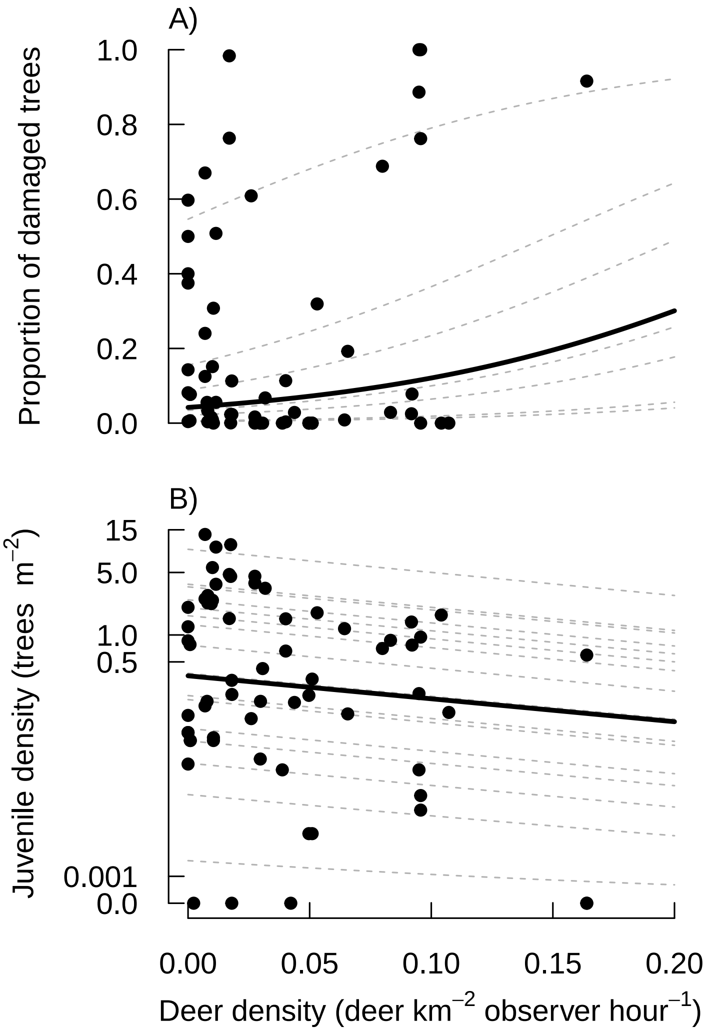

Figure 9. Relationship between deer density and the A) proportion of damaged and B) total number of *Betula* trees <3 m tall in tree transects at Creag Meagaidh National Nature Reserve, 2002 – 2008. Solid curves are mean model fit for A) and B) respectively: $\text{logit}(y) = -3.12 + 11.4x$ and $\log(y) = 4.24 - 5.95x$. The percent of deviance explained by the models in A) and B) was respectively 97.5 and 97.4% at the observation-level when random effects were considered. Dashed lines are mean relationships within individual A) years and B) transects, which accounted for most random variation in respective models (see text for details).

of juvenile birch trees declined with deer density ($\chi^2 = 7.7$, $p = 0.006$; Fig. 9B). Temporal variation was stronger than variation among measured transects for the proportion of damaged trees but the reverse was true for tree density (ratio of estimated temporal : spatial variance: 2.6 and 5.9×10^{-3}, respectively). Variance estimates for the probability of browsing ($\upsilon_j = 1.38$ and $\upsilon_k = 3.57$ were similar to those estimated for the matrix model in Table 2; $\upsilon_j = 5.41$, $\upsilon_k = 0.03$, and $\upsilon_{ijk} = 0.08$ for tree density model).

6 References

Atkinson, M. D. 1992. *Betula pendula* Roth (*B. verrucosa* Ehrh.) and *B. pubescens* Ehrh. *Journal of Ecology* **80**:837–870.

Bates, D., and M. Maechler. 2009. *lme4: Linear mixed-effects models using S4 classes. R package version 0.999375-32.*

Bee, J., A. Tanentzap, W. Lee, R. Lavers, A. Mark, J. Mills, and D. Coomes. 2008. The benefits of being in a bad neighbourhood: plant community composition influences red deer foraging decisions. *Oikos* **118**:18–24.

Bee, J., D. Wright, A. Tanentzap, W. Lee, R. Lavers, J. Mills, A. Mark, and D. Coomes. 2010. Spatio-temporal feeding selection of red deer in a mountainous landscape. *Austral Ecology* **35**:752–764.

Belisle, C. J. P. 1992. Convergence theorems for a class of simulated annealing algorithms on *rd*. *Journal of Applied Probability* **29**:885–895.

Bélisle, C. J. P. 1992. Convergence theorems for a class of simulated annealing algorithms on rd. *Journal of Applied Probability* **29**:885–895.

Bolker, B. M., M. E. Brooks, C. J. Clark, S. W. Geange, J. R. Poulsen, M. H. H. Stevens, and J. S. White. 2009. Generalized linear mixed models: a practical guide for ecology and evolution. *Trends in Ecology & Evolution* **24**:127–135.

Burnham, K. P., and D. R. Anderson. 2002. *Model selection and multimodel inference: a practical information-theoretic approach.* Springer, New York.

Canham, C. D., P. T. LePage, and K. D. Coates. 2004. A neighborhood analysis of canopy tree competition: effects of shading versus crowding. *Canadian Journal of Forest Research* **34**:778–787.

Caspersen, J. P., and M. Saprunoff. 2005. Seedling recruitment in a northern temperate forest: the relative importance of supply and establishment limitation. *Canadian Journal of Forest Research* **35**:978–989.

Clark, J. S., E. Macklin, and L. Wood. 1998. Stages and spatial scales of recruitment limitation in southern appalachian forests. *Ecological Monographs* **68**:213–235.

Coomes, D. A., R. P. Duncan, R. B. Allen, and J. Truscott. 2003. Disturbances prevent stem sizedensity distributions in natural forests from following scaling relationships. *Ecology Letters* **6**:980–989.

Elston, D. A., R. Moss, T. Boulinier, C. Arrowsmith, and X. Lambin. 2001. Analysis of aggregation, a worked example: numbers of ticks on red grouse chicks. *Parasitology* **122**:563–569.

Enquist, B. J., G. B. West, E. L. Charnov, and J. H. Brown. 1999. Allometric scaling of production and life-history variation in vascular plants. *Nature* **401**:907–911.

Gelman, A., and J. Hill. 2007. *Data analysis using regression and multilevel/hierarchical models.* Cambridge University Press, Cambridge.

Gelman, A., and I. Pardoe. 2006. Bayesian measures of explained variance and pooling in multilevel (hierarchical) models. *Technometrics* **48**:241–251.

Gill, R. M. A., and G. Morgan. 2010. The effects of varying deer density on natural regeneration in woodlands in lowland Britai. *Forestry* **83**:53–63.

Gómez-Aparicio, L., and C. D. Canham. 2008. Neighborhood models of the effects of invasive tree species on ecosystem processes. *Ecological Monographs* **78**:69–86.

Greene, D. F., C. D. Canham, K. D. Coates, and P. T. Lepage. 2004. An evaluation of alternative dispersal functions for trees. *Journal of Ecology* **92**:758–766.

Hester, A. J., J. Miles, and C. H. Gimingham. 1991. Succession from heather moorland to birch woodland. II. Growth and competition between *Vaccinium myrtillus, Deschampsia flexuosa* and *Agrostis capillaris*. *Journal of Ecology* **79**:317–327.

Kinnaird, J. W. 1974. Effect of site conditions on the regeneration of birch (*Betula* pendula Roth and *B. pubescens* Ehrh.). *Journal of Ecology* **62**:467–472.

Koda, R., and N. Fujita. 2011. Is deer herbivory directly proportional to deer population density? comparison of deer feeding frequencies among six forests with different deer density. *Forest Ecology and Management* **262**:432–439.

Koh, S., D. R. Bazely, A. J. Tanentzap, D. R. Voigt, and E. Da Silva. 2010. *Trillium grandiflorum* height is an indicator of white-tailed deer density at local and regional scales. *Forest Ecology and Management* **259**:1472–1479.

Kohyama, T., E. Suzuki, T. Partomihardjo, T. Yamada, and T. Kubo. 2003. Tree species differentiation in growth, recruitment and allometry in relation to maximum height in a Bornean mixed dipterocarp forest. *Journal of Ecology* **91**:797–806.

LePage, P. T., C. D. Canham, K. D. Coates, and P. Bartemucci. 2000. Seed abundance versus substrate limitation of seedling recruitment in northern temperate forests of British Columbia. *Canadian Journal of Forest Research* **30**:415–427.

Millett, J., A. Hester, P. Millard, and A. McDonald. 2006. How do different competing species influence the response of *Betula pubescens* Ehrh. to browsing? *Basic and Applied Ecology* **7**:123–132.

Mountford, E. P., and G. F. Peterken. 2000. Growth, mortality and regeneration in Craigellachie, a semi-natural birchwood in the Scottish Highlands. *Botanical Journal of Scotland* **52**:187–211.

Muller-Landau, H. C., R. S. Condit, K. E. Harms, C. O. Marks, S. C. Thomas, S. Bunyavejchewin, G. Chuyong, L. Co, S. Davies, R. Foster, S. Gunatilleke, N. Gunatilleke, T. Hart, S. P. Hubbell, A. Itoh, A. R. Kassim, D. Kenfack, J. V. LaFrankie, D. Lagunzad, H. S. Lee, E. Losos, J. Makana, T. Ohkubo, C. Samper, R. Sukumar, I. Sun, M. N. N. Supardi, S. Tan, D. Thomas, J. Thompson, R. Valencia, M. I. Vallejo, G. V. Muoz, T. Yamakura, J. K. Zimmerman, H. S.

Dattaraja, S. Esufali, P. Hall, F. He, C. Hernandez, S. Kiratiprayoon, H. S. Suresh, C. Wills, and P. Ashton. 2006. Comparing tropical forest tree size distributions with the predictions of metabolic ecology and equilibrium models. *Ecology Letters* **9**:589–602.

Palmer, S., A. Hester, D. Elston, I. Gordon, and S. Hartley. 2003. The perils of having tasty neighbors: grazing impacts of large herbivores at vegetation boundaries. *Ecology* **84**:2877–2890.

Plummer, M. 2011. *JAGS: just another Gibbs sampler. Version 3.0.0.*

Pounden, E., D. F. Greene, M. Quesada, and J. M. C. Sánchez. 2008. The effect of collisions with vegetation elements on the dispersal of winged and plumed seeds. *Journal of Ecology* **96**:531–598.

Putman, R., P. Duncan, and R. Scott. 2005. Demographic changes in a Scottish red deer population (*Cervus elaphus* L.) in response to sustained and heavy culling: an analysis of trends in deer populations of Creag Meagaidh National Nature Reserve 1986-2001. *Forest Ecology and Management* **206**:263–281.

R Development Core Team. 2011. *R: A Language and Environment for Statistical Computing.* R Foundation for Statistical Computing, Vienna.

Ribbens, E., J. A. Silander, and S. W. Pacala. 1994. Seedling recruitment in forests: Calibrating models to predict patterns of tree seedling dispersion. *Ecology* **75**:1794.

Ridout, M. S. 2009. Statistical applications of the complex-step method of numerical differentiation. *American Statistician* **63**:66–74.

Russo, S. E., S. W. Wiser, and D. A. Coomes. 2007. Growth-size scaling relationships of woody plant species differ from predictions of the Metabolic Ecology Model. *Ecology Letters* **10**:889–901.

Schupp, E., T. Milleron, and S. Russo. 2002. Dissemination limitation and the origin and maintenance of speciesrich tropical forests. Pages 69–82 *in* D. Levey, W. Silva, and M. Galetti, eds. *Seed Dispersal and Frugivory: Ecology, Evolution, and Conservation.* CAB International Press, Wallingford.

Silverman, B. W. 1986. *Density Estimation for Statistics and Data Analysis.* 1st ed. Chapman and Hall/CRC, Boca Raton.

Su, Y.-S., and M. Yajima. 2011. *R2jags: A Package for Running jags from R.* R package version 0.02-17.

Tremblay, J., J. Huot, and F. Potvin. 2006. Divergent nonlinear responses of the boreal forest field layer along an experimental gradient of deer densities. *Oecologia* **150**:78–88.

Ward, J. S., and G. R. Stephens. 1997. Survival and growth of yellow birch (*Betula alleghaniensis*) in southern New England. *Canadian Journal of Forest Research* **27**:156–165.

Westoby, M. 1984. The self-thinning rule. *Advances in Ecological Research* **14**:167–225.

Wyckoff, P. H., and J. S. Clark. 2000. Predicting tree mortality from diameter growth: a comparison of maximum likelihood and Bayesian approaches. *Canadian Journal of Forest Research* **30**:156–167.

Yoda, K. 1963. Self-thinning in over-crowded pure stands under cultivated and natural conditions. *Journal of Biology, Osaka City University* **14**:107–129.

7 Appendix S1 – JAGS code for matrix model

```
model
 {
for (i in 1:60){                                          # loops across each plot
 for (j in yrst[i]:yren[i]){                              # go across each time, allowing for variable lengths
  browse12[i,j] ~ dbin(dd2[i, j], total12[i,j])           # model number of browsed trees in 0 -- 2 m height tier
  # calculate probability of browsing
  logit(dd2[i,j]) <- intercept + plotvar[i] + yearr[j]
  d[i,j] <- exp(dd2[i,j]*dd)                              # calculate non-linear effect of deer

   # calculate transition probabilities for each element in A matrix
   A[i,j,1,2] <- 0
   A[i,j,1,3] <- 0
   A[i,j,2,2] <- s[2,j]-g[2,j]
   A[i,j,2,3] <- 0
   A[i,j,3,1] <- 0
   A[i,j,3,2] <- g[2,j]
   A[i,j,3,3] <- pow(1-(1-mm),2)
   A[i,j,1,1] <- d[i,j]*(s[1,j]-g[1,j])
   A[i,j,2,1] <- d[i,j]*g[1,j]

  for (k in 1:3){                                         # loop across each height tier
```

```
      treearray[i,j+1,k] ~ dpois(n1p[i,j,k])        # model observed numbers of trees at t+1
      # calculate numbers of trees at t+1 from numbers at t (=treearray) and seedling rain (= nt * se)
      n1p[i,j,k] <- exp(err[i, j, k])*((inprod(A[i,j,k,],treearray[i,j,]) + nt[i]*mb[i,j,k]))
      err[i,j,k] ~ dnorm(0.00000E+00, taue)         # add residual error
     }

   # calculate matrix b in text that normalizes total seedling rain nt
   mb[i,j,1] <- (1-meand*(means-meang))/(1-pow(meand*(means-meang), 28)) * (1 + (sr[j]-gr[j])*sqrt(d[i,j]))
   mb[i,j,2] <- 0
   mb[i,j,3] <- 0
  }
}

################################
# parameters used in calculation of seed rain
################################
means <- sqrt(ilogit(alpha[1]))         # calculate mean survival
meang <- 1 - sqrt(1 - ilogit(beta[1]))  # calculate mean growth

for (i in 1:60){                        # loop across plots
 meand[i] <- sqrt(exp(dd*ilogit(intercept + plotvar[i])))
}
```

```
 #################################
 # priors for remaining parameters
 #################################
for (i in 1:60){
  plotvar[i] ~ dnorm(0, tauplot)                 # calculate spatial variation in probability of browsing
}

# priors for matrix parameters
for (i in 1:2){                                  # loop across height tiers
 for (j in 1:4){                                 # loop across time periods
                logit(g[i,j]) <- beta[i] + vg[j]
                logit(s[i,j]) <- alpha[i] + vs[j]
               }
 alpha[i] ~ dnorm(0, .0001)
 beta[i] ~ dnorm(0, .0001)
}

for (j in 1:4){                                  # loop across time periods
        vg[j] ~ dnorm(0, taug)
        vs[j] ~ dnorm(0, taus)
        yearr[j] ~ dnorm(0, tauyearr)
}
```

```
  # set priors
  intercept ~ dnorm(0, .0001)

  tauyearr <- pow(sigyearr, -2)
  sigyearr ~ dunif(0, 100)

  tauplot <- pow(sigplot, -2)
  sigplot ~ dunif(0, 100)

  taug <- pow(taugy, -2)
  taugy ~ dunif(0, 100)

  taus <- pow(tausy, -2)
  tausy ~ dunif(0, 100)

  taue <- pow(sige, -2)
  sige ~ dunif(0, 100)

  dd ~ dnorm(0,.0001)

  mm ~ dnorm(.975,precmm)        # draw from known distribution for mortality in >3 m tier
  precmm <- pow(0.03, -2)
} #end
```
