# Supplementary material for: Getting the biggest birch for the bang: restoring and expanding upland birchwoods in the Scottish Highlands by managing red deer
Source: Ecol Evol. 2013 May 22;3(7):1890–901. doi: 10.1002/ece3.548 (PMC3728932; doi:10.1002/ece3.548)
Supplement: Supplementary file 7 [file ece30003-1890-SD7.doc]

**SUPPORTING INFORMATION**

**Getting the biggest birch for the bang: restoring and expanding upland birchwoods in the Scottish Highlands by managing red deer**

Andrew J. Tanentzap, James Zou, and David A. Coomes

**Appendix 3** – Additional methods

*Field measurements of birch population dynamics*

*Seedling censuses*: We established six plots ranging from 0.6–4.0 ha to survey birch seedlings (<2 m in height) in relation to parent trees. Plots were located within a 300 m radius of adult trees (≥3 m height) and we recorded the height and location of each adult within this distance. Within each plot, we randomly located 160–427 0.5 × 0.5 m quadrats and counted the number of seedlings and visually estimated the ground cover for the following dominant (>25% cover) vegetation types: bracken (Pteridium *aquilinum*), *Agrostis*-*Festuca* grassland, heather (*Calluna vulgaris*), moss, purple moor grass (*Molinia caerulea*), heath (*Erica* spp.), bog myrtle (*Myrica gale*), and bilberry (*Vaccinium* spp.).

*Juvenile growth and survival*: Juvenile birch trees (<3 m in height) were monitored along six permanently marked 1-km long transects that were located in areas where tree regeneration was expected. Each transect was measured in May or June every other year from 2002 to 2010. At 100 m intervals along each transect, a 100 × 2 m plot was located in which all trees emerging from unique seedlings were counted in three size classes: 0–2 m, 2–3 m, and >3 m. The number of leader stems that were visibly damaged by mammalian herbivores was recorded in each plot, i.e. broken stem, bark chewed/stripped. In November 2009, we also visually estimated the dominant vegetation ground cover types (>25% cover) within each plot and recorded the heights and locations of all adult trees within 300 m of each plot (*n =* 12 581 parent trees).

*Adult growth, allometry, and survival*: In 2008, we measured 40 adult birch trees (>3 m height) at Corrour Estate, directly south of Craeg Mageidh. Increment cores (4.3 mm diameter) were extracted at breast height, air-dried, mounted, and sanded until growth rings were visible. Annual growth was determined by measuring the width of each annual ring using digital imaging software, and we used mean annual radial growth for the previous 8-years in analyses. For each tree, we also recorded the diameter at breast height (dbh), standing height, measured with a Suunto clinometer (Suunto Oy, Vantaa, Finland), and crown diameter, estimated as the mean of two perpendicular ground-based measurements. We combined this dataset with 188 trees mapped in the Creag Meagaidh seedling plots in order to generate a size-structure for estimating mortality (see Science Manual, Supporting Information).

*Assumptions of validation procedure*

Our validation procedure made three important assumptions. Firstly, we assumed that the composition of ground cover was relatively unchanged from 1988 to 2009, and thus, 2009 measurements of ground cover could be used to predict substrate favourability. None of the transects were in areas that had been burned, and in the absence of anthropogenic disturbances, there are likely to be few changes in coarse vegetation classifications over the 21-years from when our model was initiated and substrates were measured (Hester *et al*. 1996). Since we lacked the spatial coordinates for juvenile trees that were established prior to the start of simulations, we could not consider them in our simulations. Thus, we added the number of juveniles recorded in plots in the first year of simulations to our model predictions. This makes a second assumption that there was no competition between trees already established along transects, if any, and incoming recruits. Our model might underestimate juvenile densities if seeds dispersed prior to the start of simulations remain dormant in the seed bank and emerge afterward, but *Betula* spp. are absent or extremely rare in the soils of open-hill habitats (Miller & Cummins 2003). Finally, we assumed that any disparity between predicted and observed values would accumulate minimally over the time scale of our study, so predictions from the validation after 10 years would vary as little from observed values as those from simulations after 30 years.

*References*

Hester, A.J., Miller, D.R. & Towers, W. (1996) Landscape-scale vegetation change in the Cairngorms, Scotland, 1946-1988: Implications for land management. *Biological Conservation*, **77**, 41-51.

Miller, G.R. & Cummins, R.P. (2003) Soil seed banks of woodland, heathland, grassland, mire and montane communities, Cairngorm Mountains, Scotland. *Plant Ecology*, **168**, 255-266.
