# Supplementary material for: Getting the biggest birch for the bang: restoring and expanding upland birchwoods in the Scottish Highlands by managing red deer
Source: Ecol Evol. 2013 May 22;3(7):1890–901. doi: 10.1002/ece3.548 (PMC3728932; doi:10.1002/ece3.548)
Supplement: Supplementary file 8 [file ece30003-1890-SD8.doc]

**SUPPORTING INFORMATION**

**Getting the biggest birch for the bang: restoring and expanding upland birchwoods in the Scottish Highlands by managing red deer**

Andrew J. Tanentzap, James Zou, and David A. Coomes

**Table S1**. Raw parameter estimates for each of three submodels (italicized) used in simulations. Estimates were derived from models fitted to observed field data using maximum-likelihood methods and Bayesian inference (see Science Manual, Supporting Information for details), and parameters were assumed to be normally distributed and on a linear scale, where otherwise specified.

| Parameter | Mean | SD |
| --- | --- | --- |
| *Juvenile recruitment (Section 2, Science Manual)* | | |
| *STR*, potential number of recruits produced by adult tree | 1 045.8 | 272.6 |
| *X_0_*, mode of dispersal distance function in m | 0.67 | 0.08 |
| *X_b_*, steepness of dispersal distance function | 1.81 | 0.02 |
| Relative establishment of recruits, bracken | 0.24 | 0.17 |
| Relative establishment of recruits, *Agrostis*-*Festuca* grass | 1.00 | 0.13 |
| Relative establishment of recruits, heather | 0.91 | 0.15 |
| Relative establishment of recruits, moss | <0.01 | <0.01 |
| Relative establishment of recruits, purple moor grass | 0.15 | 0.01 |
| Relative establishment of recruits, heath | 0.23 | <0.01 |
| Relative establishment of recruits, bog myrtle | 0.05 | 0.32 |
| Relative establishment of recruits, bilberry | 0.01 | 0.23 |
| *Juvenile growth and mortality (Section 3, Science Manual)* | | |
| Deer effect (coefficient for probability of browsing, logit scale) | -2.57 | 0.98 |
| Mean probability of browsing over two years (%) | 8.8 | 17.4 |
| Mean survival over two years in 0–2 m height tier (%) | 97.3 | 10.2 |
| Mean survival over two years in 2–3 m height tier (%) | 96.6 | 14.1 |
| Mean growth over two years in 0–2 m height tier (%) | 0.97 | 0.64 |
| Mean growth over two years in 2–3 m height tier (%) | 13.5 | 8.97 |
| Temporal variance in browsing over two years (logit scale) | 3.20 | 2.58 |
| Spatial variance in browsing over two years (logit scale) | 2.29 | 0.41 |
| Temporal variance in survival over two years (logit scale) | 40.0 | 27.6 |
| Temporal variance in growth over two years (logit scale) | 1.50 | 1.70 |
| *Adult growth and mortality (Section 4, Science Manual)* | | |
| Exponent for power-law height growth estimated from diameter | 0.40 | 0.05 |
| Constant for power-law height growth estimated from diameter | 9.23 | 1.28 |
| Exponent for power-law crown diameter estimated from height | 1.18 | 0.16 |
| Constant for power-law crown diameter estimated from height | 0.28 | 0.11 |
| Annual mortality | 2.48 | <0.01 |

**Table S2**. Sensitivity of model to changes in number of recruits produced by each adult tree (*STR*) and their survival in the 0–2 m height tier (*s*_1_). We re-ran the validation procedure each time by multiplying STR and/or *s*_1_ by combinations of values ranging from 0.95–1.10, and estimated the mean (95% CIs) for the regression between predicted and observed values within fifty 2 × 100 m plots at Creag Meagaidh in 2000 (as described in the main text). *D* is the proportion of deviance explained by model fit. All parameter sets produced a slope and intercept overlapping 1 and 0, respectively, so for use in simulations, we simply chose the values with a slope closest to one. Bolded values indicate parameter estimates used in final simulation model.

| Change in *STR* | Change in *s*_1_ | Intercept | Slope | *D* |
| --- | --- | --- | --- | --- |
| 0.95 | 0.95 | 3.7 (-0.7–8.2) | 1.47 (0.92–2.02) | 0.68 |
| 0.95 | 1.00 | 4.2 (-0.6–9.0) | 1.25 (0.77–1.74) | 0.65 |
| 1.00 | 0.95 | 3.8 (-0.8–8.3) | 1.48 (0.91–2.04) | 0.65 |
| 1.00 | 1.00 | 4.2 (-0.8–9.2) | 1.13 (0.67–1.59) | 0.63 |
| 0.95 | 1.05 | 4.0 (-0.9–9.0) | 1.16 (0.69–1.64) | 0.63 |
| 0.95 | 1.10 | 4.1 (-0.7–8.9) | 1.11 (0.67–1.54) | 0.62 |
| 1.00 | 1.05 | 3.6 (-0.7–7.9) | 1.09 (0.69–1.48) | 0.67 |
| 1.00 | 1.10 | 4.1 (-0.4–8.5) | 1.03 (0.65–1.41) | 0.68 |
| 1.05 | 0.95 | 4.2 (-0.9–9.2) | 1.22 (0.71–1.72) | 0.61 |
| 1.05 | 1.00 | 3.8 (-0.7–8.4) | 1.12 (0.69–1.54) | 0.65 |
| 1.05 | 1.05 | 3.9 (-0.6–8.3) | 1.10 (0.69–1.51) | 0.67 |
| 1.05 | 1.10 | 4.0 (-0.7–8.7) | 0.95 (0.58–1.32) | 0.63 |
| 1.10 | 0.95 | 4.2 (-0.7–9.0) | 1.22 (0.74–1.71) | 0.63 |
| **1.10** | **1.00** | **3.9 (-0.5–8.3)** | **1.00 (0.63–1.37)** | **0.66** |
| 1.10 | 1.05 | 3.9 (-0.6–8.3) | 0.98 (0.62–1.35) | 0.66 |
| 1.10 | 1.10 | 4.2 (-0.6–8.9) | 0.89 (0.55–1.24) | 0.65 |

**Fig. S1**. Lower and upper 95% confidence intervals (CIs) for numbers of juvenile trees in 0–2 (**top row**) and 2–3 m (**middle row**) height tiers and basal area of adult trees (>3 m tall; **bottom row**) predicted after 30 years from initial densities of 500 adult trees ha^-1^. Both substrate favourability and deer browsing varied in 10% intervals, and we performed 100 simulations at each combination of these two factors (*n* = 121). 95% CIs for tree counts calculated as for a Poisson distributed variable:
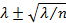
, where *λ* is the mean and *n* is the number of observations.

**Fig. S2**. Lower and upper 95% confidence intervals (CIs) for numbers of juvenile trees in 0–2 (**top row**) and 2–3 m (**middle row**) height tiers and basal area of adult trees (>3 m tall; **bottom row**) predicted after 30 years from initial densities of 250 adult trees ha^-1^. Both substrate favourability and deer browsing varied in 10% intervals, and we performed 100 simulations at each combination of these two factors (*n* = 121). 95% CIs for tree counts calculated as for a Poisson distributed variable:
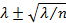
, where *λ* is the mean and *n* is the number of observations.

**Fig. S3**. Lower and upper 95% confidence intervals (CIs) for numbers of juvenile trees in 0–2 (**top row**) and 2–3 m (**middle row**) height tiers and basal area of adult trees (>3 m tall; **bottom row**) predicted after 30 years from initial densities of 500 adult trees ha^-1^. Trees were randomly located in ten 0.1 ha patches (i.e. “patch invasion model”). Both substrate favourability and deer browsing varied in 10% intervals, and we performed 100 simulations at each combination of these two factors (*n* = 121). 95% CIs for tree counts calculated as for a Poisson distributed variable:
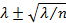
, where *λ* is the mean and *n* is the number of observations.

**Fig. S4**. Predicted responses of juvenile tree regeneration in 0–2 and 2–3 m height tiers along gradients of deer browsing and substrate favourability. Both deer browsing and substrate favourability varied in 10% intervals, and we performed 100 simulations at each combination of these two factors (total *n* = 12 100). Simulations were repeated at initial adult densities of 250 and 500 trees ha^-1^ with the “core invasion” and “patch invasion” models (only 500 trees ha^-1^ used for the latter model; see main text for model details). Solid line is model fit in the absence of active management, and at mean initial adult density (= 417 trees ha^-1^), and (**a**, **c**) mean substrate favourability (= 50%); or (**b**, **d**) mean deer density (= 50%). Equations of lines: (**a**) *y* = *e*^13.41 – 0.83^*^x^*; (**b**) *y* = *e*^13.41 – 0.61^*^x^*; (**c**) *y* = *e*^9.52 – 1.52^*^x^*; (**d**) *y* = *e*^9.52 – 0.53^*^x^*.
